# Association Between Fixed-Dose Combination Use and Medication Adherence, Health Care Utilization, and Costs Among Medicaid Beneficiaries

**DOI:** 10.1016/j.jacadv.2025.102091

**Published:** 2025-08-21

**Authors:** Donglan Zhang, Jun Soo Lee, Nicole L. Therrien, Lisa M. Pollack, Sandra L. Jackson, Xiaobei Dong, Anand Rajan, Kai Hong, Andrew E. Moran, Feijun Luo

**Affiliations:** a Center for Population Health and Health Services Research, Department of Foundations of Medicine, New York University Grossman Long Island School of Medicine, Mineola, New York, USA; b Department of Population Health, New York University Grossman School of Medicine, New York, New York, USA; c Division for Heart Disease and Stroke Prevention, Centers for Disease Control and Prevention, Atlanta, Georgia, USA; d Joseph J. Zilber College of Public Health, University of Wisconsin-Milwaukee, Milwaukee, Wisconsin, USA; e Policy Analysis and Engagement Office, Office of Policy, Performance, and Evaluation, Centers for Disease Control and Prevention, Atlanta, Georgia, USA; f Division of General Medicine, Columbia University Irving Medical Center, New York, New York, USA

**Keywords:** combination-pill therapy, fixed-dose combination, hypertension management, medication adherence, Medicaid beneficiaries, out-of-pocket costs

## Abstract

**Background:**

Fixed-dose combination (FDC) antihypertensives combine two or more agents. Compared with non-FDC antihypertensives of multiple classes (multi-pill therapy), combination-pill therapy using FDC antihypertensives may improve hypertension control. However, combination-pill therapy remains low.

**Objectives:**

This study aims to assess: 1) the association between combination-pill therapy and medication adherence, health care utilization, and costs; and 2) the potential to mitigate racial and ethnic differences in medication adherence.

**Methods:**

A retrospective cohort analysis was conducted using the 2017-2021 Merative MarketScan Medicaid database. The study sample included adults aged 18 to 64 years with hypertension, continuously enrolled 1 year before and after a random index date of prescription. The propensity score overlap weighting method was used to balance characteristics between individuals using combination- and multi-pill therapy. Logistic models were used for medication adherence (defined as medication possession ratio [MPR] ≥80%), linear models for continuous MPRs, negative binomial models for health care utilization, and generalized linear models for costs.

**Results:**

Compared with multi-pill therapy, combination-pill therapy was associated with higher medication adherence (3.17 in MPR; 95% CI: 2.79-3.55), fewer hypertension-related emergency department visits (220 per 1,000 individuals; 95% CI: −235 to −204), fewer hospitalizations (153 per 1,000 individuals, 95% CI: −160 to −146), and lower costs ($2,862 per person, 95% CI: −$3,035 to −$2,689). However, differences in medication adherence persisted, with non-Hispanic Black adults demonstrating lower adherence than non-Hispanic White adults.

**Conclusions:**

Combination-pill therapy could improve hypertension management and save costs for the Medicaid program and beneficiaries. However, persistent racial and ethnic differences in adherence highlight the need for tailored interventions.

Hypertension is a major risk factor for cardiovascular disease (CVD), stroke, kidney disease, and dementia,^1^, ^2^, ^3^ affecting about half of Americans based on the 2017 American College of Cardiology and American Heart Association (ACC/AHA) definition for hypertension (threshold: 130/80 mm Hg).^4^ Non-Hispanic Black (NHB) adults experience a disproportionately higher burden of hypertension, with substantially higher prevalence, lower medication adherence, and lower rates of blood pressure control than non-Hispanic White (NHW) and Hispanic adults.^5^^,^^6^

Fixed-dose combination (FDC) therapy that combines ≥2 antihypertensive medications into a single pill (a treatment strategy referred to as combination-pill therapy in this study) simplifies medication regimens and has been proven safe and effective.^7^ Combination-pill therapy using FDC antihypertensives may improve medication adherence and hypertension control.^8^ The 2017 ACC/AHA guideline recommended lower blood pressure thresholds and using combination-pill antihypertensive therapy as an initial management strategy for some individuals to control hypertension more quickly and effectively.^9^ The 2018 European Society of Cardiology/European Society of Hypertension and the Japanese Society of Hypertension guidelines also highlighted the importance of FDC antihypertensives for hypertension treatment and recommend initial combination-pill therapy in almost all patients.^10^

Despite the promise of combination-pill therapy, its utilization for hypertension treatment in the United States remains low, and its potential to address racial and ethnic differences in hypertension management has not been well studied.^11^^,^^12^ This could be due to several reasons. One is that certain FDC antihypertensives, although approved, may not be included in health care systems’ treatment protocols and insurance coverage formularies.^13^^,^^14^ Therapeutic inertia may also play a role, and there are concerns among health care professionals regarding the safety and efficacy, availability of different combinations, insurance coverage, and access to detailed FDC-antihypertensive information.^15^ These barriers may particularly affect low-income and racial and ethnic minority populations, who may disproportionately face financial burden if insurance does not cover these drugs.^16^^,^^17^ Evidence is lacking regarding whether combination-pill therapy is effective in mitigating racial and ethnic differences in hypertension management.

To address this evidence gap, using large-scale claims data, we conducted an analysis among Medicaid beneficiaries to investigate: 1) the association between combination-pill therapy and subsequent medication adherence, health care utilization and medical expenses over a 1-year period; and 2) whether the associations between combination-pill therapy and hypertension-related outcomes vary across racial and ethnic groups.

## Methods

### Data sources and study population

Data were obtained from the MerativeTM MarketScan multistate Medicaid claims database from 2016 to 2022. In accordance with the Health Insurance Portability and Accountability Act Privacy Rule that governs confidentiality and data protection, no identifiable private information was accessed, and Institutional Review Board review was not required. A data use agreement was executed between our institution and Merative. To build our study sample (Supplemental Figure 1), we first identified Medicaid beneficiaries who had ≥1 hypertension diagnosis (International Classification of Diseases (ICD)-10-CM codes I10-I15) and had ≥1 pharmacy claims for an antihypertensive medication between January 2017 and December 2021 (study period, Supplemental Table 1A). During this period, we selected a random date as the index date to define the time when individuals filled or refilled antihypertensive medications. The random selection of the index date is preferred over identifying an index date based on the order of a pharmacy claim, as the latter would oversample individuals from earlier years compared with later years.^18^ A random index date allows for a more even distribution of individuals who initiated medications at different times across the study period and has been widely used in claims data analysis.^18^^,^^19^ We then restricted the sample to those who: 1) were aged 18 to 64 years at the index date; 2) were continuously enrolled in one Medicaid insurance plan for the entire 1-year follow-up period postindex date; and 3) had 1 year of data for prescribed antihypertensives before and after the index date.

We excluded beneficiaries aged ≥65 years who may have been dually eligible for Medicare, as well as those who had a pregnancy diagnosis during the study period, as pregnancy may affect medication regimens.^20^ In addition, to streamline the comparison between those using combination-pill therapy and those using non-FDC antihypertensives for 2 different medication classes (referred to as multi-pill therapy in this study), we excluded beneficiaries who switched between combination-pill therapy and multi-pill therapy. We also excluded those who used only one antihypertensive therapeutic class to create comparable groups for the 2 therapy types. Finally, when calculating the annual health care costs, we identified a subset of adults not covered by capitated health insurance plans using the capitation indicator and plan type variables because fixed annual payment model plans do not fully reflect health care costs incurred when receiving services.^21^

## Outcomes

The primary outcomes of the study included: 1) antihypertensive medication adherence within 1-year postindex date; 2) emergency department (ED) visits and inpatient admissions associated with hypertension and CVD per 1,000 individuals within 1-year postindex date; 3) hypertension-related medical costs (overall and separately for ED visits and inpatient admissions) within 1-year postindex date; and 4) CVD-related medical costs (overall and separately for ED visits and inpatient admissions) within 1-year postindex date. Sensitivity analyses examined all-cause costs. Hypertension-related and CVD-related health care utilization and total medical costs were defined based on the presence of the respective diagnosis codes (hypertension: ICD-10-CM codes I10-I15; CVD: ICD-10-CM codes I00-I78).^22^

Medication adherence was measured using the medication possession ratio (MPR), with MPR ≥80% indicating adequate adherence to antihypertensive medications.^23^^,^^24^ To be aligned with the structure of the claims data and consistent with our previous analyses,^25^^,^^26^ we defined medication adherence using the MPR. The average MPR was computed as the ratio of the total days of antihypertensive drug supply to the number of days in each year.^23^ For combination-pill therapy, we derived the average MPRs for all available FDC antihypertensives in the database (Supplemental Table 1B) and for the 7 antihypertensive therapeutic classes including angiotensin-converting enzyme (ACE) inhibitors, angiotensin receptor blockers (ARBs), beta-blockers, calcium-channel blockers (CCB), diuretics, other antihypertensives, and renin-angiotensin system antagonists separately, along with the corresponding generic drug names.^24^ For multi-pill therapy, we derived the average MPRs for each antihypertensive medication. MPR was chosen over metrics like proportion of days covered (PDC) because it provides a more comprehensive view of medication availability—particularly for multidrug regimens or FDCs, as seen in recent antihypertensive studies^27^^,^^28^—while PDC may underestimate adherence due to its conservative day-level coverage.

For our secondary outcomes, we assessed: 1) Medicaid insurance payments (including all adjusted payments for health care services from the Medicaid program); 2) patient out-of-pocket (OOP) costs (including copay, coinsurance, and deductibles); and 3) total costs (the sum of the Medicaid insurance payments and patient OOP costs for combination- and multi-pill therapy per individual. All secondary outcomes reflect paid pharmacy claims within 1-year postindex date.

All outcomes were stratified by race and ethnicity (NHW, NHB, and Hispanic populations). Race and ethnicity information was obtained directly from the MarketScan Medicaid Database enrollment records. Following the MarketScan classification system, individuals were categorized as NHW, NHB, Hispanic, and other racial groups. According to the MarketScan user guide, “other racial groups” included Asian, Pacific Islander, American Indian/Alaska Native, and those reporting multiple races. There were no missing race/ethnicity data in our analytical sample. We did not stratify by other racial groups due to their high heterogeneity.

### Exposure

Combination-pill therapy was defined as having a pharmacy claim for ≥1 FDC-antihypertensive medication and no other antihypertensive medications from the index date through the follow-up period. The comparison was multi-pill therapy, defined as having no claims for FDC antihypertensives but having claims for ≥2 different antihypertensive medication classes from the index date through the follow-up period.

### Statistical analysis

We used propensity score (PS) overlap weighting methods to balance characteristics between individuals using combination- and multi-pill therapy (Supplemental Figure 2).^29^^,^^30^ In calculating the PS, we included age groups (18-34, 35-44, 45-54, and 55-64 years), sex, race and ethnicity (NHW, NHB, Hispanic, and other racial groups), dummy indicators for risk factors (alcohol use, tobacco use, obesity, and lipid disorders), and 12 comorbidities identified in the Charlson comorbidity index (excluding myocardial infarction, congestive heart failure, peripheral vascular disease, cerebrovascular disease, and renal disease from the original Charlson comorbidity index because these comorbidities may be in the causal pathways between antihypertensive medication use and CVD outcomes).^31^ All sociodemographic and comorbidity variables were measured in the 1-year period prior to the index date. We conducted detailed balance diagnostics using standardized mean differences before and after applying PS overlap weighting to assess the adequacy of our adjustment strategy. The values <0.1 indicate adequate balance.^32^ We employed logistic models for medication adherence and linear models for MPRs. We then employed negative binomial models to estimate all health care utilization outcomes. We utilized generalized linear models with gamma distribution and log link function to accommodate the skewed distribution of health care costs.^33^ In sensitivity analyses, we included those who used both combination-pill and multi-pill therapy in the same year (to create a more inclusive definition of the combination-pill therapy group, allowing for inclusion in the exposure group of patients who may have switched between regimens or who may have been taking an FDC antihypertensive along with additional antihypertensive agents simultaneously) and tested the robustness of the results.

All main analyses were conducted using the overlap-weighted cohorts. All models were additionally adjusted for fixed effects for the year and month of the index date to control for time-invariant fixed effects within a year or a month. In each model, we compared outcomes between combination- and multi-pill therapy groups within the entire population, as well as within each race and ethnicity. We tested whether outcomes between combination- and multi-pill therapy groups differed by race/ethnicity by examining the statistical significance of interaction terms between therapy type and race/ethnicity.

Average marginal effects and corresponding 95% CIs were reported. Sensitivity analyses were conducted to assess the association between combination-pill therapy and all-cause health care utilization, as combination-pill therapy may have spillover effects on other disease outcomes and related health care utilization. All analyses were conducted using Stata SE 17.0 statistical software (StataCorp) and SAS version 9.4 (SAS 9.4, SAS Institute Inc).

## Results

After sample selection, a total of 325,600 adults with Medicaid coverage were included in the analysis of health care utilization outcomes. In the subset analysis among those not covered by capitated insurance, 128,528 adults were included in the assessment of cost outcomes (Supplemental Figure 1).

### Sample characteristics

Among 325,600 adults, 29,328 (9%) used combination-pill therapy to manage their hypertension, while 296,272 (91%) used multi-pill therapy (Table 1). Compared with the multi-pill therapy group, the combination-pill therapy group was significantly younger (48.2 vs 49.4; *P* < 0.001) and had a higher proportion of females (64.11% vs 55.01%; *P* < 0.001) and NHB adults (33.12% vs 28.15%; *P* < 0.001). The combination-pill therapy group also had a lower proportion of alcohol and tobacco use (both *P* < 0.001) and a lower proportion of some comorbidities (dementia, chronic pulmonary disease, rheumatic disease, and diabetes), but higher proportion of others (obesity, diabetes, and lipid disorders) (all *P* < 0.001). After weighting the sample using the PS overlap approach, the characteristics between individuals using combination-pill and multi-pill therapy were well-balanced (standardized mean difference <0.1), indicating that the 2 groups were comparable at baseline (Supplemental Figure 2, Supplemental Table 2).

**Table 1:** Summary Statistics of Sample Characteristics

Characteristics	All (N = 325,600, 100%)	Combination-Pill Therapy (n = 29,328, 9%)	Multi-Pill Therapy (n = 296,272, 91%)	*P* Value^a^
Age, y, mean (SD)	49.3 (10.1)	48.2 (9.9)	49.4 (10.2)	<0.001
Age groups, y, n (%)				<0.001
18-34	31,428 (9.65%)	2,884 (9.83%)	28,544 (9.63%)	
35-44	67,531 (20.74%)	7,415 (25.28%)	60,116 (20.29%)	
45-54	102,795 (31.57%)	9,537 (32.52%)	93,258 (31.48%)	
55-64	123,846 (38.04%)	9,492 (32.36%)	114,354 (38.60%)	
Female, n (%)	181,776 (55.83%)	18,802 (64.11%)	162,974 (55.01%)	<0.001
Race categories, n (%)				<0.001
Non-Hispanic White	174,306 (53.53%)	14,527 (49.53%)	159,779 (53.93%)	
Non-Hispanic Black	93,122 (28.60%)	9,712 (33.12%)	83,410 (28.15%)	
Hispanic	13,736 (4.22%)	1,290 (4.40%)	12,446 (4.20%)	
Other race groups	44,436 (13.65%)	3,799 (12.95%)	40,637 (13.72%)	
Risk factors, n (%)				
Alcohol use	21,338 (6.55%)	867 (2.96%)	20,471 (6.91%)	<0.001
Tobacco use	83,496 (25.64%)	5,160 (17.59%)	78,336 (26.44%)	<0.001
Obesity	79,341 (24.37%)	6,273 (21.39%)	73,068 (24.66%)	<0.001
Lipid disorders	103,085 (31.66%)	6,769 (23.08%)	96,316 (32.51%)	<0.001
Comorbidities, n (%)				
Dementia	1,530 (0.47%)	49 (0.17%)	1,481 (0.50%)	<0.001
Chronic pulmonary disease	71,344 (21.91%)	4,052 (13.82%)	67,292 (22.71%)	<0.001
Rheumatic disease	6,811 (2.09%)	433 (1.48%)	6,378 (2.15%)	<0.001
Peptic ulcer disease	2,353 (0.72%)	97 (0.33%)	2,256 (0.76%)	<0.001
Mild liver disease	19,666 (6.04%)	734 (2.50%)	18,932 (6.39%)	<0.001
Diabetes without chronic complication	102,364 (31.44%)	6,054 (20.64%)	96,310 (32.51%)	<0.001
Diabetes with chronic complication	40,848 (12.55%)	1,452 (4.95%)	39,396 (13.30%)	<0.001
Hemiplegia or paraplegia	4,729 (1.45%)	140 (0.48%)	4,589 (1.55%)	<0.001
Any malignancy	10,139 (3.11%)	662 (2.26%)	9,477 (3.20%)	<0.001
Moderate or severe liver disease	3,071 (0.94%)	26 (0.09%)	3,045 (1.03%)	<0.001
Metastatic solid tumor	1,731 (0.53%)	96 (0.33%)	1,635 (0.55%)	<0.001
AIDS/HIV	3,154 (0.97%)	242 (0.83%)	2,912 (0.98%)	0.009

a We employed the Wilcoxon nonparametric rank sum test to examine differences in means across continuous variables, while Pearson's chi-square test was utilized to assess variations in proportions across categorical variables based on combination-pill and multi-pill therapies.

### Association between combination-pill therapy and medication adherence

After PS overlap weighting, compared with multi-pill therapy, combination-pill therapy was associated with, on average, an 8.5 percentage point (95% CI: 7.9-9.1) higher likelihood of medication adherence (MPR ≥80%) or 3.17 (95% CI: 2.79-3.55) higher average MPR (Table 2). The associations were strongest among NHW adults, who had a 9.7 percentage point (95% CI: 8.9-10.5) higher likelihood of medication adherence when using combination-pill vs multi-pill therapy, compared with the 6.9 percentage point (95% CI: 5.9-7.9) higher likelihood among NHB and 5.7 percentage point (95% CI: 2.9-8.5) among Hispanic adults (interaction *P* <0.001).

**Table 2:** The Association of Combination-Pill Therapy Use With Medication Adherence and Medication Possession Ratios (per Individual)^a^

Medication Adherence	All	NH White	NH Black	Hispanic
Medication adherence (MPR ≥80%) to antihypertensives				
Multi-pill therapy^b^	0.395	0.444	0.309	0.386
	(0.393-0.397)	(0.442-0.447)	(0.305-0.312)	(0.376-0.395)
Combination-pill therapy only^b^	0.480	0.541	0.378	0.442
	(0.474-0.485)	(0.534-0.549)	(0.368-0.387)	(0.416-0.469)
Difference^c^	**0.085∗∗∗**	**0.097∗∗∗**	**0.069∗∗∗**	**0.057∗∗∗**
	**(0.079-0.091)**	**(0.089-0.105)**	**(0.059-0.079)**	**(0.029-0.085)**
Observations	325,600	174,306	93,122	13,736
Medication possession ratios				
Multi-pill therapy	65.66	69.23	59.27	66.33
	(65.53-65.78)	(69.06-69.40)	(59.04-59.50)	(65.72-66.93)
Combination-pill therapy only	68.83	73.11	61.03	69.94
	(68.47-69.19)	(72.60-73.63)	(60.40-61.67)	(68.26-71.63)
Difference	**3.17∗∗∗**	**3.88∗∗∗**	**1.76∗∗∗**	**3.62∗∗∗**
	**(2.79-3.55)**	**(3.34-4.42)**	**(1.09-2.44)**	**(1.83-5.40)**
Observations	325,600	174,306	93,122	13,736

NH = non-Hispanic; MPR = medication possession ratio.

a Logistic regression was employed to analyze medication adherence, while linear regression was used for medication possession ratios. Average marginal effects, along with 95% CIs, were reported. All models were adjusted for 12 Charlson comorbidities, risk factors (alcohol use, tobacco use, obesity, and lipid disorders), sex, age groups (aged 18-34, aged 35-44, aged 45-54, and aged 55-64 years), race/ethnicity, and fixed effects for the year and month of the index date.

b Those who used combination-pill therapy comprise individuals who exclusively used FDC antihypertensives during the 1-year follow-up periods from the antihypertensive index date. Those who used multi-pill therapy comprise individuals who did not use FDC antihypertensives during the 1-year follow-up periods.

c The reported differences reflect the predicted outcomes, specifically the average marginal effects, for individuals receiving combination-pill therapy compared with those receiving multi-pill therapy.

### Association between combination-pill therapy and health care utilization and costs

Compared with multi-pill therapy, those using combination-pill therapy experienced, on average, 220 fewer hypertension-related ED visits (95% CI: −235 to −204), 153 fewer hypertension-related inpatient admissions (95% CI: −160 to −146), 270 fewer CVD-related ED visits (95% CI: −287 to −254), and 181 fewer CVD-related inpatient admissions (95% CI: −189 to −173) per 1,000 individuals 1 year after the index date (Table 3). These effects were most pronounced among NHB adults. Compared with multi-pill therapy, combination-pill therapy use among NHB adults was associated with reductions of 287 (95% CI: −318 to −256) hypertension-related ED visits, 192 (95% CI: −204 to −180) hypertension-related inpatient visits, 337 (95% CI: −368 to −305) CVD-related ED visits, and 215 (95% CI: −228 to −202) CVD-related inpatient visits per 1,000 individuals 1 year after the index date. Among NHW adults, combination-pill therapy was associated with reductions of 172 (95% CI: −192 to −152) hypertension-related ED visits, 134 (95% CI: −142 to −125) hypertension-related inpatient visits, 225 (95% CI: −246 to −204) CVD-related ED visits, and 167 (95% CI: −177 to −158) CVD-related inpatient visits per 1,000 individuals 1 year after the index date. Combination-pill therapy was associated with the least reduction in ED and inpatient visits among Hispanic adults.

**Table 3:** The Association of Combination-Pill Therapy Use With Hypertension- and Cardiovascular Disease-Related Health Care Utilization (per 1,000 Individuals)^a^

Population Groups	Num. HTN-Related^c^ ED Visits	Num. HTN-Related Inpatient Admissions	Num. CVD-Related^d^ ED Visits	Num. CVD-Related Inpatient Admissions
All				
Multi-pill therapy^b^	697.7	227.5	768.1	261.2
	(689.0-706.4)	(221.6-233.3)	(758.8-777.5)	(254.3-268.0)
Combination-pill therapy only^b^	478.1	74.78	498.0	80.33
	(465.0-491.2)	(70.91-78.65)	(484.6-511.4)	(76.23-84.43)
Difference^c^	**−219.6∗∗∗**	**−152.7∗∗∗**	**−270.1∗∗∗**	**−180.9∗∗∗**
	**(−235.4 to −203.8)**	**(−159.8 to −145.6)**	**(−286.5 to −253.8)**	**(−188.8 to −172.9)**
Observations	325,600	325,600	325,600	325,600
Non-Hispanic White				
Multi-pill therapy	589.4	207.4	662.5	246.3
	(579.9-599.0)	(201.2-213.6)	(652.2-672.9)	(238.9-253.7)
Combination-pill therapy only	417.5	73.64	437.9	79.27
	(400.3-434.7)	(67.97-79.31)	(420.3-455.6)	(73.28-85.26)
Difference	**−171.9∗∗∗**	**−133.8∗∗∗**	**−224.6∗∗∗**	**−167.0∗∗∗**
	**(−192.0 to −151.8)**	**(−142.2 to −125.4)**	**(−245.5 to −203.8)**	**(−176.6 to −157.5)**
Observations	174,306	174,306	174,306	174,306
Non-Hispanic Black				
Multi-pill therapy	898.1	270.5	969.5	299.6
	(882.0-914.1)	(261.2-279.8)	(952.6-986.5)	(289.2-310.0)
Combination-pill therapy only	610.8	78.38	633.0	84.55
	(584.3-637.4)	(71.01-85.74)	(605.9-660.0)	(76.72-92.37)
Difference	**−287.2∗∗∗**	**−192.1∗∗∗**	**−336.6∗∗∗**	**−215.0∗∗∗**
	**(−318.0 to −256.4)**	**(−204.0 to −180.2)**	**(−368.3 to −304.9)**	**(−227.9 to −202.1)**
Observations	93,122	93,122	93,122	93,122
Hispanic				
Multi-pill therapy	512.9	142.8	566.9	164.4
	(488.2-537.6)	(131.8-153.8)	(540.7-593.1)	(152.2-176.6)
Combination-pill therapy only	390.3	54.48	402.7	55.87
	(324.2-456.5)	(37.93-71.03)	(334.6-470.8)	(39.01-72.74)
Difference	−122.6∗∗∗	−88.3∗∗∗	−164.2∗∗∗	−108.5∗∗∗
	**(−192.4 to −52.8)**	**(−108.5 to −68.2)**	**(−236.3 to −92.2)**	**(−129.6 to −87.5)**
Observations	13,736	13,736	13,736	13,736

**Bold** values indicate ∗*P* < 0.05; ∗∗*P* < 0.01; ∗∗∗*P* < 0.001.

CVD = cardiovascular disease; ED = emergency department; HTN = hypertension.

a A negative binomial model was used for all count variables. Average marginal effects, along with 95% CIs, were reported. All models were adjusted for 12 Charlson comorbidities, risk factors (alcohol use, tobacco use, obesity, and lipid disorders), sex, age groups (aged 18-34, aged 35-44, aged 45-54, and aged 55-64 years), race/ethnicity, and fixed effects for the year and month of the index date.

b Those who used combination-pill therapy comprise individuals who exclusively used FDC antihypertensive during the 1-year follow-up periods from the antihypertensive index date. Those who used multi-pill therapy comprise those who did not use FDC antihypertensive during the 1-year follow-up periods.

c The reported differences reflect the predicted outcomes, specifically the average marginal effects, for individuals receiving combination-pill therapy compared with those receiving multi-pill therapy.

d Hypertension (HTN)-related emergency department (ED) visits and inpatient admissions are defined as those containing an HTN diagnosis (ICD-10-CM codes I10–I15). Cardiovascular disease (CVD)-related ED visits and inpatient admissions are defined as those containing a CVD diagnosis (ICD-10-CM codes I00–I78).

Among those with noncapitated Medicaid plans, compared with multi-pill therapy, combination-pill therapy was associated with a $2,862 per-person reduction in total hypertension-related medical costs 1 year after the index date (95% CI: −$3,035 to −$2,689), including $225 (95% CI: −$251 to −$199) for hypertension-related ED visits and $2,280 (95% CI: −$2,464 to −$2,096) for hypertension-related inpatient admissions (Table 4). Combination-pill therapy was also associated with a reduction of total CVD-related medical costs by $3,779 per person (95% CI: -$3,978 to -$3,581), with reductions of $269 (95% CI: -$295 to -$242) for CVD-related ED visits, and $2,798 (95% CI: -$3,018 to -$2,578) for CVD-related inpatient visits. Among NHB adults, combination-pill therapy was associated with a reduction of hypertension-related medical costs by $3,896 per-person (95% CI: −$4,198 to −$3,595), compared with $2,126 (95% CI: −$2,339 to −$1,912) for NHW adults and $1,259 (95% CI: -$1,584 to -$934) for Hispanic adults (interaction *P* value <0.001). In addition, combination-pill therapy was associated with a reduction of CVD-related medical costs by $4,914 per-person (95% CI: −$5,258 to −$4,571) among NHB adults, compared with $3,012 (95% CI: −$3,255 to −$2,769) for NHW adults and $1,727 (95% CI: −$2,095 to −$1,359) for Hispanic adults (interaction *P* value <0.001).

**Table 4:** The Association of Combination-Pill Therapy Use With Medical Costs Associated With Hypertension and Cardiovascular Disease (per Individual)^a^

Population Groups	HTN-Related Total Medical Costs, $	HTN-Related ED Costs, $	HTN-Related Inpatient Costs, $	CVD-Related Total Medical Costs, $	CVD-Related ED Costs, $	CVD-Related Inpatient Costs, $
All						
Multi-pill therapy^b^	4,842	470	3,067	5,976	520.8	3,680
	(4,717-4,968)	(450.9-489.9)	(2,897-3,237)	(5,821-6,130)	(499.8-541.9)	(3,466-3,893)
Combination-pill therapy only^b^	1,980	245	787	2,196	252	882
	(1,858-2,102)	(228-263)	(696-878)	(2,064-2,329)	(235-270)	(781-982)
Difference^c^	**−2,862∗∗∗**	**−225∗∗∗**	**−2,280∗∗∗**	**−3,779∗∗∗**	**−269∗∗∗**	**−2,798∗∗∗**
	**(−3,035 to −2,689)**	**(−251 to −199)**	**(−2,464 to −2,096)**	**(−3,978 to −3,581)**	**(−295 to −242)**	**(−3,018 to −2,578)**
Observations	128,528	128,528	128,528	128,528	128,528	128,528
Non-Hispanic White						
Multi-pill therapy	3,900	379	2,604	4,976	436	3,264
	(3,765-4,034)	(360.2-397.2)	(2,422-2,785)	(4,809-5,144)	(416-457)	(3,035-3,492)
Combination-pill therapy only	1,774	208	855	1,964	216	969
	(1,603-1,944)	(185-232)	(705-1,005)	(1,781-2,147)	(192-240)	(804-1,134)
Difference	**−2,126∗∗∗**	**−171∗∗∗**	**−1,749∗∗∗**	**−3,012∗∗∗**	**−220∗∗∗**	**−2,294∗∗∗**
	**(−2,339 to −1,912)**	**(−200 to −141)**	**(−1,972 to −1,526)**	**(−3,255 to −2,769)**	**(−252 to −189)**	**(−2,559 to −2,030)**
Observations	60,139	60,139	60,139	60,139	60,139	60,139
Non-Hispanic Black						
Multi-pill therapy	6,235	625	3,606	7,498	679	4,202
	(5,996-6,474)	(593-657)	(3,336-3,876)	(7,214-7,783)	(645-713)	(3,877-4,527)
Combination-pill therapy only	2,339	327	693	2,584	335	783
	(2,152-2,526)	(293-360)	(575-811)	(2,378-2,790)	(302-369)	(649-916)
Difference	**−3,896∗∗∗**	**−299∗∗∗**	**−2,913∗∗∗**	**−4,914∗∗∗**	**−344∗∗∗**	**−3,419∗∗∗**
	**(−4,198 to −3,595)**	**(−344 to −253)**	**(−3,210 to −2,616)**	**(−5,258 to −4,571)**	**(−391 to −297)**	**(−3,764 to −3,074)**
Observations	39,326	39,326	39,326	39,326	39,326	39,326
Hispanic						
Multi-pill therapy	2,035	107	1,605	2,563	118	1,964
	(1,827-2,244)	(80-135)	(1,372-1,837)	(2,300-2,827)	(90-146)	(1,683-2,245)
Combination-pill therapy only	776.4	43	423	837	45	447
	(531.4-1,021)	(25-61)	(210-636)	(583-1,091)	(27-63)	(229-665)
Difference	**−1,259∗∗∗**	**−64∗∗∗**	**−1,181∗∗∗**	**−1,727∗∗∗**	**−73∗∗∗**	**−1,517∗∗∗**
	**(−1,584 to −934)**	**(−97 to −32)**	**(−1,502 to −861)**	**(−2,095 to −1,359)**	**(−106 to −40)**	**(−1,876 to −1,159)**
Observations	8,690	8,690	8,690	8,690	8,690	8,690

Abbreviations as in Table 3.

a Hypertension-related and CVD-related health care utilization and total medical costs were defined based on the presence of respective diagnosis codes (hypertension: ICD-10-CM codes I10-I15; CVD: ICD-10-CM codes I00-I78). A generalized linear model with a gamma distribution and log link was used for all cost outcomes. Average marginal effects, along with 95% CIs, were reported. All models were adjusted for 12 Charlson comorbidities, risk factors (alcohol use, tobacco use, obesity, and lipid disorders), sex, age groups (aged 18-34, aged 35-44, aged 45-54, and aged 55-64 years), race/ethnicity, and fixed effects for the year and month of the index date.

b Those who used combination-pill therapy comprise individuals who exclusively used FDC antihypertensives during the 1-year follow-up periods from the antihypertensive index date. Those who used multi-pill therapy comprise those who did not use FDC antihypertensive during the 1-year follow-up periods.

c The reported differences reflect the predicted outcomes, specifically the average marginal effects, for individuals receiving combination-pill therapy compared with those receiving multi-pill therapy.

### Sensitivity analyses

In sensitivity analyses, compared with multi-pill therapy, combination-pill therapy was associated with a significant reduction in all-cause health care utilization and medical costs 1 year after the index date, with a stronger association observed among NHB adults (*P* < 0.001) (Supplemental Tables 3 and 4). Furthermore, 82,126 (22%) used both combination- and multi-pill therapy during the same year, (Supplemental Table 5). Including individuals who used both therapies did not alter the findings or conclusions (Supplemental Figure 3, Supplemental Tables 6 to 9). To ensure transparency, we also presented all unweighted results in Supplemental Tables 10 to 12.

### Comparison of annual and out-of-pocket costs between combination- and multi-pill therapy groups

**Figure 1:**
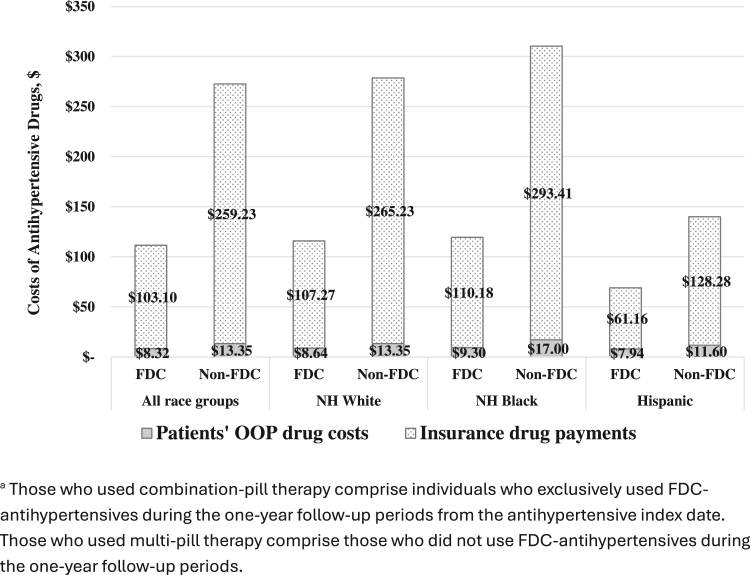
**Insurance and Patients’ Out-of-Pocket Costs** Comparison of payment between combination-pill therapy and multi-pill therapy by racial and ethnic groups, MarketScan Medicaid, 2016-2022^a^. OOP = out-of-pocket; FDC = fixed-dose combination; NH = non-Hispanic.

The annual per-person costs for combination-pill therapy ($111 95% CI: $107-$115) were significantly lower than those for multi-pill therapy ($273, 95% CI: $270-$275). The patient OOP costs for combination-pill therapy ($8) were also lower than those for multi-pill therapy ($13) (*P* < 0.001) (Figure 1). However, among those who used combination-pill therapy, NHB beneficiaries had similar annual medication costs ($110) to NHW ($107) but significantly higher than Hispanic adults ($61).

## Discussion

In this cohort study of Medicaid beneficiaries with hypertension, we found that 9% used combination-pill therapy to manage their blood pressure. Combination-pill therapy was significantly associated with higher medication adherence, fewer ED visits and hospitalizations, and lower medical costs compared with multi-pill therapy. Combination-pill therapy was also associated with lower Medicaid payments and patient OOP costs than multi-pill therapy. Importantly, our findings revealed significant racial and ethnic differences in these associations. Although improvement in medication adherence was smaller among NHB adults than NHW adults for combination-pill therapy vs multi-pill therapy, NHB adults showed a more pronounced association with reduced health care utilization and costs. These results highlight persistent inequities in how different racial and ethnic groups experience and benefit from FDC therapy (Central Illustration).

**Central Illustration:**
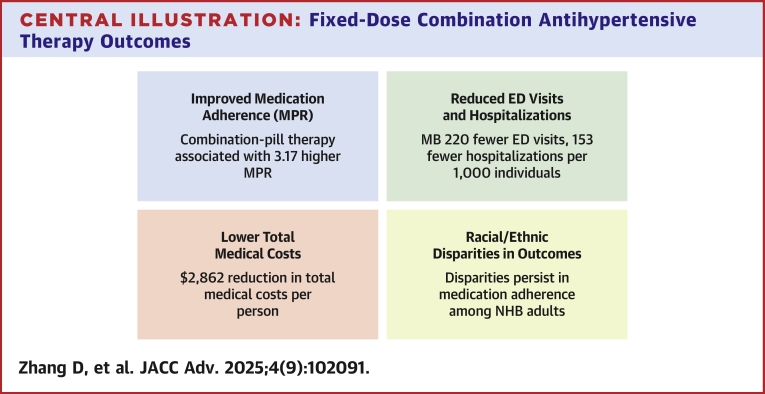
**Fixed-Dose Combination Antihypertensive Therapy Outcomes** ED = emergency department; MB = mean benefit; MPR = medication possession ratio; NHB = non-Hispanic Black.

Compared with the reported prevalence of FDC-antihypertensive/combination-pill therapy among all U.S. adults with hypertension based on National Health and Nutrition Examination Survey 2017-March 2020 (27.8%),^9^ we found a lower prevalence of Medicaid beneficiaries using FDC antihypertensives exclusively (9%, our main analysis definition of combination-pill therapy) or using a mix of FDC antihypertensives and other antihypertensives during the same year (22%; our more inclusive sensitivity analysis definition of combination-pill therapy) between 2017 and 2021. Previous reports indicated a decrease in FDC-antihypertensive use from 2012 to 2020,^9^ with Medicare and Medicaid FDC claims decreasing from 2016 to 2020.^34^ The majority of FDC antihypertensives are available in generic form,^34^ and our analysis showed that Medicaid payments and patient OOP costs were lower for combination-pill therapy. Given the small proportion of Medicaid beneficiaries with hypertension using combination-pill therapy, barriers to use of combination-pill therapy may not be attributed to costs. Prior work has documented barriers including lack of sufficiently diverse FDC-antihypertensive therapeutic options with respect to medication classes and dosage; one study estimated that nearly two-thirds of U.S. adults with hypertension were using a regimen not available as an FDC-antihypertensive product.^11^

Consistent with the literature,^35^^,^^36^ our analysis demonstrated that combination-pill therapy was associated with significantly higher medication adherence. However, combination-pill therapy was less associated with medication adherence in NHB adults (1.76; 95% CI: 1.09-2.44) compared with NHW adults (3.88; 95% CI: 3.34-4.42). This racial difference in adherence outcomes highlights structural and clinical gaps in antihypertensive care for NHB adults. This finding may be attributable to several reasons. First, the majority of FDC antihypertensives were combinations of ACE inhibitors or ARBs plus thiazide diuretics,^34^ and ACE inhibitors and ARBs may be insufficient for improving hypertension control for NHB adults, possibly due to poorer medication adherence and/or differences in physiological pathways.^37^, ^38^, ^39^ The AHA/ACC guideline recommends initial antihypertensive treatment with a thiazide diuretic or CCBs for NHB adults. However, there are no CCB/thiazide diuretic FDC antihypertensives, which represents a gap for guideline-recommended combination-pill therapy for NHB adults, who may have a less robust response to ACEIs, ARBs, and beta-blockers.^40^ Second, there may be differences in plan enrollment and coverage formularies at the state or plan level, particularly in states with managed care organizations. As shown in our analysis, there were some slight variations in costs for NHB beneficiaries compared with NHW and Hispanic beneficiaries. NHB beneficiaries may be more likely to be enrolled in plans with higher cost-sharing responsibilities compared with NHW and Hispanic Medicaid beneficiaries.^41^ However, our data did not include data on insurance benefit designs, so this is beyond the scope of the present analysis and may be investigated in national Medicaid claims databases in the future.

We observed that combination-pill therapy was associated with greater reductions in ED and inpatient visits among NHB adults compared with NHW adults, despite a smaller association with medication adherence. This could be because NHB adults initially had much lower medication adherence and higher health care utilization and costs. Thus, they might benefit more from using combination-pill therapy. If NHB adults had medication adherence comparable to NHW adults, the reduction in health care utilization and costs might have been even larger. In contrast, Hispanic adults had higher medication adherence than NHB adults initially, but the lowest health care utilization and costs. Previous studies have reported that Hispanic adults are less likely to seek health care due to language, cultural barriers, and immigration status.^42^ This could partially explain why the associations between combination-pill therapy and lower health care utilization and costs were smaller among Hispanic adults compared with NHW adults. Together, these findings emphasize the importance of tailoring antihypertensive strategies to the needs of racially and ethnically diverse populations and addressing the underlying systemic factors that contribute to differences in hypertension outcomes.

Our study has important implications. While combination-pill therapy has the potential to improve medication adherence, the available options in the United States may not offer the variety of class and dose combinations preferred by clinicians. An analysis of 2015-2020 National Health and Nutrition Examination Survey data found 189 unique therapeutic class combination regimens among U.S. adults using antihypertensives, only 7 of which (3.7%) were available as FDC antihypertensives.^11^ Similar findings are seen among combination regimens in the SPRINT (Systolic Blood Pressure Intervention Trial).^11^^,^^43^ There are no available combinations containing 4 antihypertensives, which were used by over 25% of SPRINT participants. The availability of only two- or three-drug FDC antihypertensives, and no four- or five-drug FDC antihypertensives, limits the treatment options for patients in the United States who require multiple antihypertensives to achieve hypertension control.^44^ Furthermore, the need for triple and quadruple drug combinations may increase because ACC/AHA hypertension treatment guidelines have recommended more intensive treatment goals (lower target blood pressures).^45^ Factors such as drug shortages, changes in insurance coverage or cost-sharing tiers, or concerns about side effects may deter initiation or prompt switching from FDC antihypertensives to other antihypertensives. Moreover, while 90% of Medicaid plans cover at least four FDC antihypertensives, and 100% cover at least one FDC-antihypertensive, individual drug coverage and costs vary by state Medicaid programs.^44^ Some drugs are on preferred (Tier 1) or nonpreferred (Tier 2) tiers, which may influence clinicians' prescribing behavior. To address these gaps, policymakers and pharmaceutical companies could consider exploring and broadening FDC-antihypertensive options with a wider range of drug class combinations to suit the variety of treatment needs faced by prescribing clinicians.^46^ Policymakers and pharmaceutical developers must also consider how the availability—or lack—of FDC options that are appropriate for NHB and Hispanic adults may contribute to persistent racial and ethnic differences in hypertension control.

### Study Limitations

We acknowledge several limitations. First, due to the use of a random index date rather than a new-user design, our study population includes both incident and prevalent users of antihypertensive medications. This approach, while enhancing representativeness and minimizing temporal bias, introduces the potential for prevalent user bias, as individuals already stabilized on therapy may differ in unmeasured ways from new users in terms of adherence behavior, clinical stability, or health care utilization patterns. Although we addressed this concern through robust PS overlap weighting to achieve covariate balance between treatment groups, we acknowledge that residual confounding due to prior medication history may persist. Future studies employing new-user designs could further delineate treatment initiation effects from long-term adherence patterns. Second, we only tracked short-term outcomes over a single year of combination-pill therapy use, leaving the long-term impacts of combination-pill therapy, such as mortality and productivity, unknown. Third, as we used a multistate Medicaid claims database, our findings may not be generalizable to individuals covered by other types of insurance, particularly because the price of FDC antihypertensives covered by Medicaid tends to be lower than that of equivalent drugs covered by Medicare and private insurance.^34^ Additionally, individual drug coverage and costs vary by state Medicaid programs, which may lead to differences in access and treatment patterns across different states. However, due to confidentiality restrictions, the MarketScan Medicaid Database lacks geographic identifiers, limiting our ability to account for state-level differences in formularies, single-pill combinations’ availability, and prescribing patterns. Fourth, our claims database only shows whether prescriptions were filled and picked up, not if patients actually took the medications. In addition, we could not analyze data at the prescribing clinician level, accounting for variance due to clinician behavior. These issues may lead to potentially overestimation of medication adherence. Fifth, our main analysis focused on individuals exclusively using combination-pill therapy (excluding those who used both combination-pill therapy and other antihypertensives in a single year), which may limit generalizability. However, our sensitivity analyses included individuals who used both combination- and multi-pill therapy in the same year, and results were consistent with the main findings. Also, we could not ascertain the doses of FDC antihypertensives, which may affect the health care utilization outcomes we assessed. Finally, our use of MPR rather than PDC as an adherence metric has important limitations. MPR may overestimate adherence compared to PDC, particularly in cases of overlapping fills or early refills. While PDC is commonly used in quality metrics such as star ratings, we chose MPR based on the claims data structure and our previous analytical approaches.^25^^,^^26^ Future studies comparing these metrics in the context of FDC-antihypertensive use would be valuable.

## Conclusions

This large-scale study among Medicaid beneficiaries in multiple states (5-8 states from year to year) across the United States found that combination-pill therapy was associated with higher medication adherence, lower health care utilization, and lower health care costs for the Medicaid program and beneficiaries. However, the magnitude of these associations differs by racial and ethnic groups. These research findings may help inform policies related to FDC-antihypertensive medication coverage or formulary decisions, as well as programs aimed at improving medication adherence and hypertension control. Future efforts should prioritize targeted strategies to ensure that combination-pill therapy reach all racial and ethnic groups. In addition, our findings can provide important evidence for clinicians when selecting antihypertensive medications for patients.

Perspectives **COMPETENCY IN MEDICAL KNOWLEDGE:** FDC therapy is associated with improved medication adherence, reduced ED visits and hospitalizations, and lower health care costs among Medicaid beneficiaries with hypertension. However, differences in adherence persist, particularly among nonHispanic Black adults, highlighting the need for more inclusive and effective therapeutic options and tailored clinical care.

**TRANSLATIONAL OUTLOOK:** Despite clinical guidelines recommending FDC therapy for hypertension management, its adoption remains low in the U.S. Medicaid population. Expanding the availability of FDC antihypertensives that align with guideline-recommended regimens—especially for populations with historically lower adherence—may enhance hypertension control and reduce health care utilization. Future research should explore long-term outcomes and the impact of policy changes on FDC uptake and hypertension control.

## Funding support and author disclosures

The findings and conclusions in this report are those of the authors and do not necessarily represent the official position of the Centers for Disease Control and Prevention. Use of trade names and commercial sources is for identification only and does not imply endorsement by the U.S. Department of Health and Human Services. The authors have reported that they have no relationships relevant to the contents of this paper to disclose.
