# Supplementary material for: Association Between Fixed-Dose Combination Use and Medication Adherence, Health Care Utilization, and Costs Among Medicaid Beneficiaries
Source: JACC Adv. 2025 Aug 21;4(9):102091. doi: 10.1016/j.jacadv.2025.102091 (PMC12398786; doi:10.1016/j.jacadv.2025.102091)
Supplement: Supplemental Data [file mmc1.docx]

**Supplemental Figure 1. Study Sample Selection, MarketScan® Medicaid Database, 2016-2022**

Medicaid enrollees with at least one hypertension diagnosis (ICD-10-CM codes I10-I15) who were prescribed at least one antihypertensive medication between January 2017 and December 2021 (n=1,400,348)

Not continuously enrolled during -364 days and +364 days from the index date (n=725,597 [52%])

Continuously enrolled during -364 days and +364 days from the randomly selected index date of antihypertensives (n=674,751 [48%])

Pregnancy diagnosis during -364 days and +364 days from the index date (n=39,871 [6%])

No pregnancy diagnosis during -364 days and +364 days from the index date (n=634,880 [94%])

Individuals aged <18 or aged >64 (n=49,395 [8%])

Individuals aged 18-64 at the index date (n=585,485 [92%])

Individuals who had no claims on FDC and had less than two therapeutic classes of antihypertensive (n=207,087 [33%])

Individuals who had either a fixed-dose combination (FDC) or at least two different therapeutic classes of antihypertensives (n=378,398 [67%])

Individuals who had both FDC and non-FDC antihypertensives (n=52,798 [14%])

Excluding individuals who had both FDC and non-FDC antihypertensives (n=**325,600** [86%])

Note: This represents the final sample for health care utilization outcomes.

Capitated health insurance during -364 days and +364 days from the index date (n=197,072 [61%])

Non-capitated health insurance: -364 days and +364 days from the index date (n= **128,528** [39%])

Note: This is the final sample for cost outcomes.

**Supplemental Figure 2. Distribution of Combination-Pill Therapy and Multi-Pill Therapy Uses in Unweighted and Overlap-Weighted Samples^a^**

**A: Final sample for healthcare utilization outcomes (N = 325,600)**


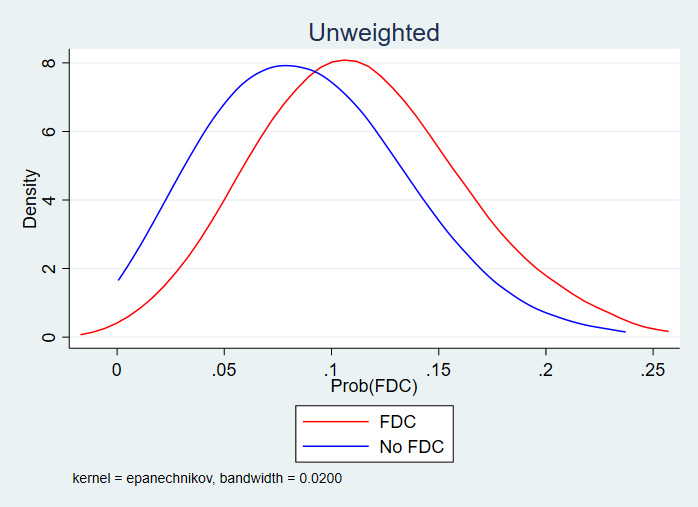

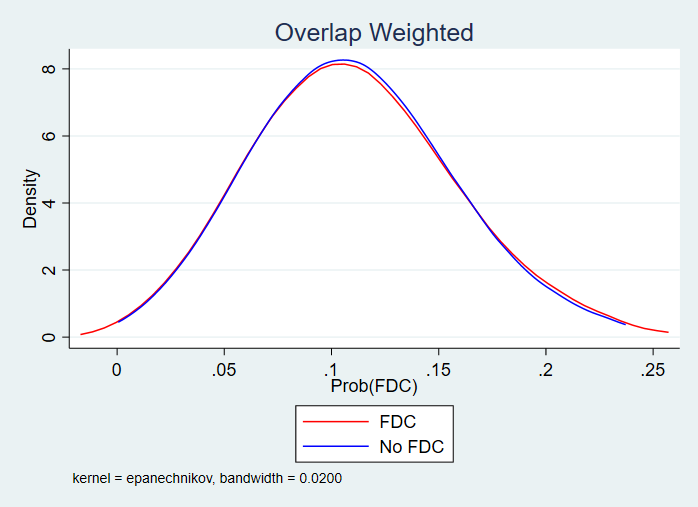


**B: Final sample for cost outcomes (N=128,528)**


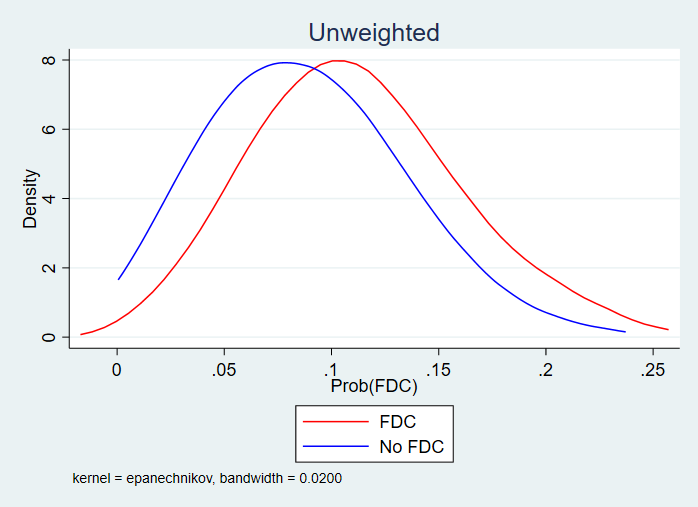

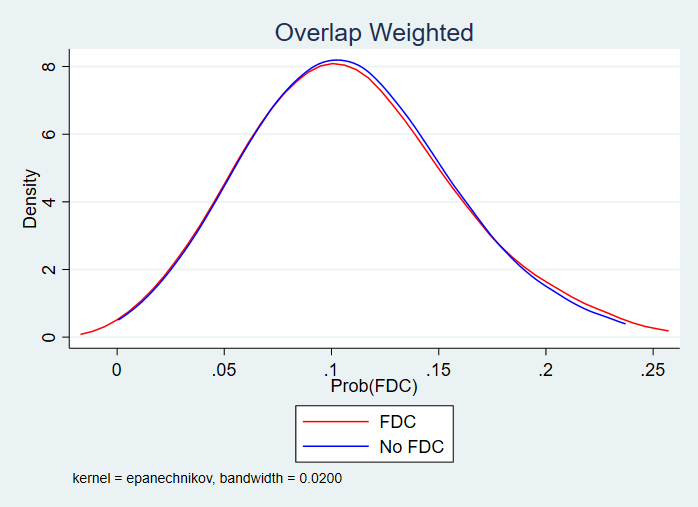


**C: Final sample for healthcare utilization outcomes for sensitivity analysis (N = 378,398)**

**
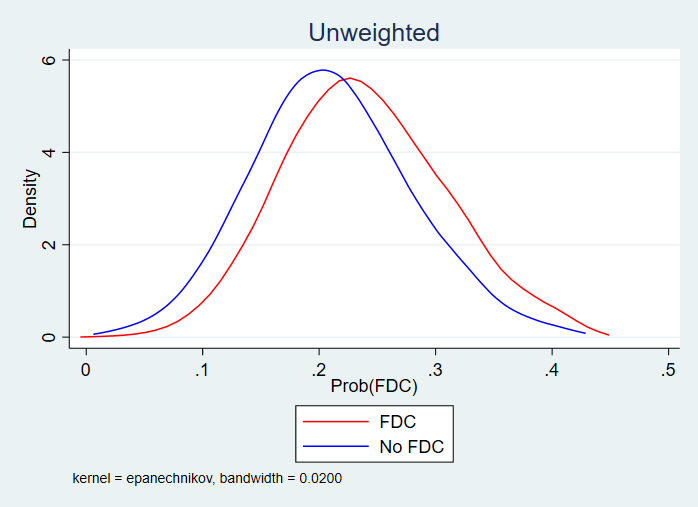

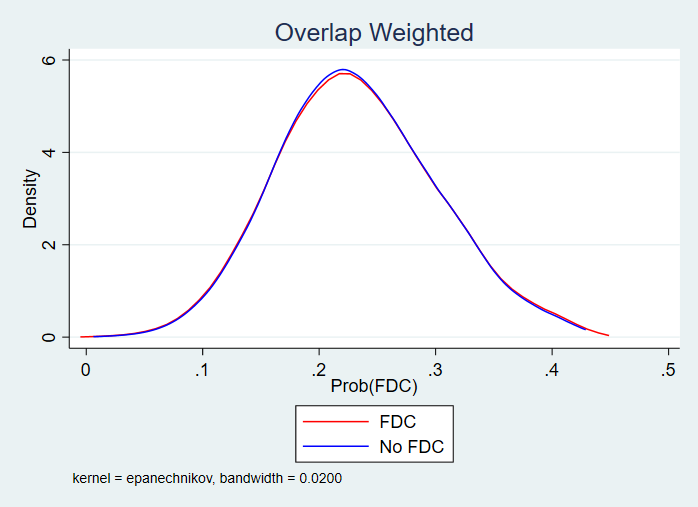
**

**D: Final sample for cost outcomes for sensitivity analysis (N=148,121)
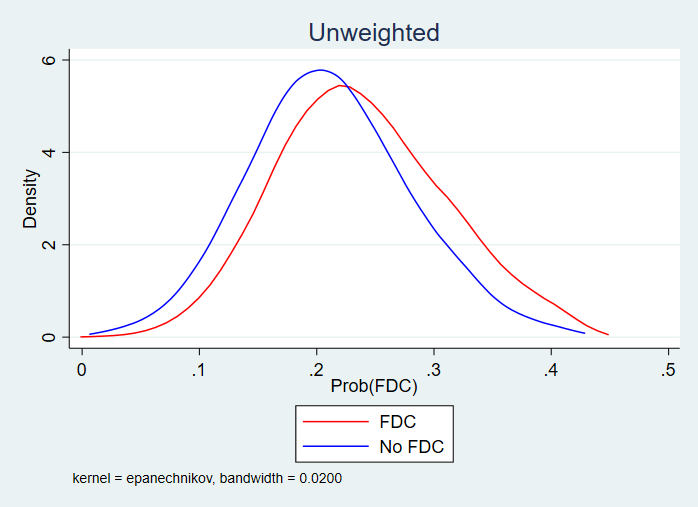
**^
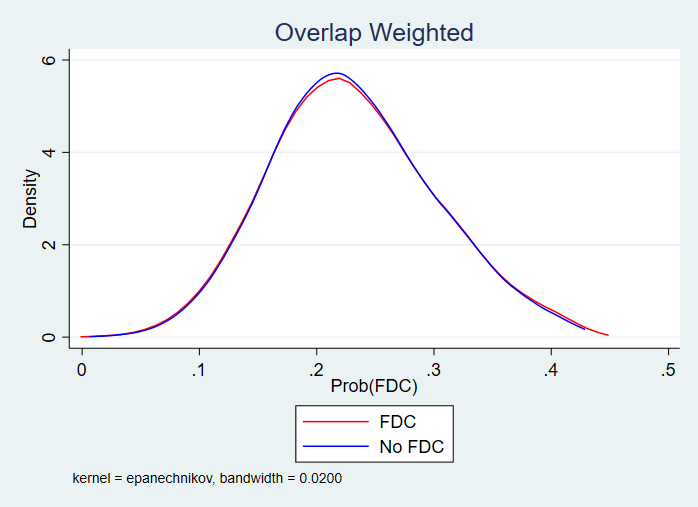
^

^a^ The probability was estimated from a logit model. The figures present the estimated probability distributions of FDC-antihypertensive use in both unweighted and overlap-weighted samples, categorized by FDC indicator. The overlapping probability distribution in the overlap-weighted sample supports the validity of the propensity-score overlap method used in this study. In Panels A and B, individuals using combination-pill therapy comprise those who exclusively used FDC-antihypertensive during the one-year follow-up periods from the antihypertensive index date. Individuals using multi-pill therapy comprise those who did not use FDC-antihypertensives during the one-year follow-up periods. In panels C and D, individuals using combination-pill therapy comprise individuals who used any FDC-antihypertensives during the one-year follow-up periods from the antihypertensive index date. Individuals using multi-pill therapy comprise those who did not use FDC-antihypertensives during the one-year follow-up periods.

**Supplemental Figure 3. Sensitivity Analysis: Sample Selection Flow Chart Including Both Combination-Pill and Multi-Pill Therapy Users**

Medicaid enrollees with at least one hypertension diagnosis (ICD-10-CM codes I10-I15) who were prescribed at least one antihypertensive medication between January 2017 and December 2021 (n=1,400,348)

Not continuously enrolled during -364 days and +364 days from the index date (n=725,597 [52%])

Continuously enrolled during -364 days and +364 days from the randomly selected index date of antihypertensives (n=674,751 [48%])

Pregnancy diagnosis during -364 days and +364 days from the index date (n=39,871 [6%])

No pregnancy diagnosis during -364 days and +364 days from the index date (n=634,880 [94%])

Individuals aged <18 or aged >64 (n=49,395 [8%])

Individuals aged 18-64 at the index date (n=585,485 [92%])

Individuals who had no FDC and had less than two therapeutic classes of antihypertensive (n=207,087 [33%])

Individuals who had either a fixed-dose combination (FDC) or at least two different therapeutic classes of antihypertensives (n=**378,398** [67%])

Note: This represents the final sample for health care utilization outcomes.

Capitated health insurance during -364 days and +364 days from the index date (n=230,277 [61%])

Non-capitated health insurance: -364 days and +364 days from the index date (n= **148,121** [39%])

Note: This is the final sample for cost outcomes.

**Supplemental Table 1A. Generic drug list for antihypertensives by therapeutic class**

| Therapeutic Class | Antihypertensive Medications |
| --- | --- |
| ACE inhibitor | Benazepril  Bepridil  Captopril  Enalapril  Fosinopril  Lisinopril  Moexipril  Perindopril  Quinapril  Ramipril  Trandolapril |
| Angiotensin receptor blocker | Azilsartan  Candesartan  Eprosartan  Irbesartan  Losartan  Olmesartan  Telmisartan  Valsartan |
| Beta blocker | Acebutolol  Atenolol  Betaxolol  Bisoprolol  Carvedilol  Labetalol  Metoprolol succinate  Metoprolol tartrate  Nadolol  Nebivolol  Pindolol  Propranolol |
| Calcium channel blocker | Amlodipine  Diltiazem  Felodipine  Isradipine  Levamlodipine  Nicardipine  Nifedipine  Nisoldipine  Verapamil |
| Diuretic | Amiloride  Bumetanide  Chlorothiazide  Chlorthalidone  Furosemide  Hydrochlorothiazide  Indapamide  Methyclothiazide  Metolazone  Torsemide  Triamterene |
| Renin-angiotensin system antagonists | Aliskiren  Azilsartan  Benazepril  Bepridil  Candesartan  Captopril  Enalapril  Eprosartan  Fosinopril  Irbesartan  Lisinopril  Losartan  Moexipril  Olmesartan  Perindopril  Quinapril  Ramipril  Telmisartan  Trandolapril  Valsartan |
| Other antihypertensives | Clonidine  Doxazosin  Eplerenone  Guanabenz  Guanfacine  Hydralazine  Methyldopa  Minoxidil  Prazosin  Spironolactone  Terazosin |

Cite: Lee, J. S., Segura Escano, R., Therrien, N. L., Kumar, A., Bhatt, A., Pollack, L. M., ... & Luo, F. (2024). Antihypertensive Medication Adherence and Medical Costs, Health Care Use, and Labor Productivity Among People With Hypertension. Journal of the American Heart Association, 13(21), e037357.

**Supplemental Table 1B. Generic drugs and combinations used to identify fixed-dose combinations**

| Generic Drug 1 | Generic Drug 2 | Generic Drug 3 |
| --- | --- | --- |
| Aliskiren | Amlodipine Besylate |  |
| Aliskiren | Amlodipine Besylate | Hydrochlorothiazide |
| Aliskiren | Hydrochlorothiazide |  |
| Aliskiren | Valsartan |  |
| Amiloride Hydrochloride | Hydrochlorothiazide |  |
| Amlodipine | perindopril Arginine |  |
| Amlodipine Besylate | Benazepril Hydrochloride |  |
| Amlodipine Besylate | HCTZ | Olmesartan Medoxomil |
| Amlodipine Besylate | Hydrochlorothiazide | Valsartan |
| Amlodipine Besylate | Olmesartan Medoxomil |  |
| Amlodipine Besylate | Telmisartan |  |
| Amlodipine Besylate | Valsartan |  |
| Atenolol | Chlorthalidone |  |
| Azilsartan Medoxomil | Chlorthalidone |  |
| Benazepril Hydrochloride | Hydrochlorothiazide |  |
| Bendroflumethiazide | Nadolol |  |
| Bisoprolol Fumarate | hydrochlorothiazide |  |
| Candesartan Cilexetil | Hydrochlorothiazide |  |
| Captopril | Hydrochlorothiazide |  |
| Chlorothiazide | Methyldopa |  |
| Chlorothiazide | Reserpine |  |
| Chlorthalidone | Clonidine Hydrochloride |  |
| Chlorthalidone | Reserpine |  |
| Deserpidine | Hydrochlorothiazide |  |
| Deserpidine | Methyclothiazide |  |
| Diltiazem Malate | Enalapril Maleate |  |
| Enalapril Maleate | Felodipine |  |
| Enalapril Maleate | Hydrochlorothiazide |  |
| Eprosartan Mesylate | Hydrochlorothiazide |  |
| Fosinopril Sodium | Hydrochlorothiazide |  |
| Guanethidine Monosulfate | Hydrochlorothiazide |  |
| HCTZ | Hydralazine HCl | Reserpine |
| Hydralazine Hydrochloride | Hydrochlorothiazide |  |
| Hydralazine Hydrochloride | Reserpine |  |
| Hydrochlorothiazide | Moexipril Hydrochloride |  |
| Hydrochlorothiazide | Olmesartan Medoxomil |  |
| Hydrochlorothiazide | Propranolol Hydrochloride |  |
| Hydrochlorothiazide | Quinapril Hydrochloride |  |
| Hydrochlorothiazide | Reserpine |  |
| Hydrochlorothiazide | Spironolactone |  |
| Hydrochlorothiazide | Labetalol Hydrochloride |  |
| Hydrochlorothiazide | Lisinopril |  |
| Hydrochlorothiazide | Losartan Potassium |  |
| Hydrochlorothiazide | Methyldopa |  |
| Hydrochlorothiazide | Metoprolol Succinate |  |
| Hydrochlorothiazide | Metoprolol Tartrate |  |
| Hydrochlorothiazide | Telmisartan |  |
| Hydrochlorothiazide | Timolol Maleate |  |
| Hydrochlorothiazide | Triamterene |  |
| Hydrochlorothiazide | Valsartan |  |
| Hydrochlorothiazide | Irbesartan |  |
| Methyclothiazide | Reserpine |  |
| Nebivolol | valsartan |  |
| Polythiazide | Prazosin Hydrochloride |  |
| Trandolapril | verapamil Hydrochloride |  |

**Supplemental Table 2. Assessment of Balance of Baseline Characteristics between FDC and Non-FDC Groups Before and After Propensity Score Overlap Weighting**

|  | SMD^a^ Before Propensity Score Overlap Weighting | SMD^a^ After Propensity Score Overlap Weighting |
| --- | --- | --- |
| Age | -0.112 | 0.005 |
| Age groups |  |  |
| 18-34 | 0.007 | 0.000 |
| 35-44 | 0.119 | 0.000 |
| 45-54 | 0.022 | -0.000 |
| 55-64 | -0.131 | -0.000 |
| Female | 0.186 | 0.000 |
| Race categories |  |  |
| Non-Hispanic White | -0.088 | -0.000 |
| Non-Hispanic Black | 0.108 | 0.000 |
| Hispanic | 0.010 | -0.000 |
| Other race groups | -0.022 | -0.000 |
| Risk factors |  |  |
| Alcohol use | -0.183 | -0.000 |
| Tobacco use | -0.215 | -0.000 |
| Obesity | -0.078 | -0.000 |
| Lipid disorders | -0.212 | 0.000 |
| Comorbidities |  |  |
| Dementia | -0.058 | -0.000 |
| Chronic pulmonary disease | -0.232 | -0.000 |
| Rheumatic disease | -0.051 | -0.000 |
| Peptic ulcer disease | -0.058 | -0.000 |
| Mild liver disease | -0.189 | -0.000 |
| Diabetes without chronic complication | -0.271 | -0.000 |
| Diabetes with chronic complication | -0.293 | -0.000 |
| Hemiplegia or paraplegia | -0.107 | -0.000 |
| Any malignancy | -0.058 | -0.000 |
| Moderate or severe liver disease | -0.126 | -0.000 |
| Metastatic solid tumor | -0.034 | -0.000 |
| AIDS/HIV | -0.017 | -0.000 |

^a^ Standardized mean difference

**Supplemental Table 3. The association of combination-pill therapy uses with all-cause health care utilization (per 1,000 individuals) using a negative binomial model^a^**

|  | Num. ED visits | Num. Inpatient Admissions | Avg. LOS | Total LOS |
| --- | --- | --- | --- | --- |
| All |  |  |  |  |
| Multi-pill therapy ^b^ | 1,442 | 304.1 | 917.1 | 1,884 |
|  | (1,428 - 1,455) | (296.2 - 312.0) | (884.3 - 949.9) | (1,788 - 1,980) |
| Combination-pill therapy ^b^ | 964.5 | 94.56 | 282.9 | 413.9 |
|  | (943.7 - 985.2) | (89.94 - 99.18) | (261.5 - 304.3) | (379.0 - 448.9) |
| Difference ^c^ | **-477.2***** | **-209.5***** | **-634.2**** | **-1469.8***** |
|  | **(-502.3 to -452.1)** | **(-218.5 to -200.6)** | **(-669.4 to -599.0)** | **(-1560.7 to -1378.9)** |
| Observations | 325,600 | 325,600 | 325,600 | 325,600 |
| Non-Hispanic White |  |  |  |  |
| Multi-pill therapy | 1,364 | 299.2 | 894.2 | 1,811 |
|  | (1,347 - 1,381) | (290.3 - 308.2) | (858.1 - 930.4) | (1,709 - 1,913) |
| Combination-pill therapy | 902.2 | 95.24 | 274.0 | 401.0 |
|  | (874.1 - 930.4) | (88.48 - 102.0) | (247.6 - 300.5) | (356.7 - 445.3) |
| Difference | **-461.8***** | **-204.0***** | **-620.2***** | **-1409.9***** |
|  | **(-494.8 to -428.8)** | **(-215.0 to -192.9)** | **(-663.3 to -577.1)** | **(-1511.8 to -1308.1)** |
| Observations | 174,306 | 174,306 | 174,306 | 174,306 |
| Non-Hispanic Black |  |  |  |  |
| Multi-pill therapy | 1,633 | 331.8 | 1,026 | 2,181 |
|  | (1,609 - 1,657) | (320.4 - 343.2) | (975.2 - 1,076) | (2,044 - 2,317) |
| Combination-pill therapy | 1,119 | 97.64 | 327.4 | 480.8 |
|  | (1,078 - 1,160) | (88.94 - 106.3) | (281.7 - 373.1) | (409.7 - 551.9) |
| Difference | **-513.6***** | **-234.2***** | **-698.39***** | **-1699.9***** |
|  | **(-561.3 to -466.0)** | **(-248.3 to -220.0)** | **(-761.6 to -635.2)** | **(-1843.7 to -1556.2)** |
| Observations | 93,122 | 93,122 | 93,122 | 93,122 |
| Hispanic |  |  |  |  |
| Multi-pill therapy | 1,212 | 193.9 | 630.3 | 1,108 |
|  | (1,165 - 1,259) | (179.9 - 207.9) | (558.9 - 701.7) | (974.3 - 1,242) |
| Combination-pill therapy | 850.7 | 66.94 | 197.0 | 268.3 |
|  | (755.7 - 945.7) | (48.33 - 85.54) | (119.1 - 274.9) | (162.7 - 374.0) |
| Difference | **-361.6***** | **-127.0***** | **-433.3***** | **-840.0***** |
|  | **(-466.9 to -256.4)** | **(-150.3 to -103.6)** | **(-538.7 to -327.9)** | **(-1008.3 to -671.8)** |
| Observations | 13,736 | 13,736 | 13,736 | 13,736 |

^a^ A negative binomial model was used for all count variables. Average marginal effects, along with 95% confidence intervals, were reported. All models were adjusted for 12 Charlson comorbidities, risk factors (alcohol use, tobacco use, obesity, and lipid disorders), gender, age groups (aged 18-34, aged 35-44, aged 45-54, and aged 55-64), race/ethnicity, and fixed effects for the year and month of the index date.

^b^ Individuals using combination-pill therapy comprise individuals who exclusively used FDC-antihypertensives during the one-year follow-up periods from the antihypertensive index date. Individuals using multi-pill therapy comprise those who did not use FDC-antihypertensives during the one-year follow-up periods.

^c^ The reported differences reflect the predicted outcomes, specifically the average marginal effects, for individuals receiving combination-pill therapy compared with those receiving multi-pill therapy.

*** *P*<0.001, ** *P*<0.01, * *P*<0.05

**Supplemental Table 4. The association of combination-pill therapy use with all-cause medical costs (per individual)^a^**

|  | Total medical costs, $ | ED costs, $ | Inpatient costs, $ | Outpatient costs, $ | Pharmacy prescription costs, $ |
| --- | --- | --- | --- | --- | --- |
| All |  |  |  |  |  |
| Multi-pill therapy ^b^ | 22,311 | 964.5 | 4,406 | 8,926 | 8,091 |
|  | (21,954 - 22,669) | (940.2 - 988.9) | (4,158 - 4,653) | (8,753 - 9,098) | (7,813 - 8,368) |
| Combination-pill therapy ^b^ | 12,768 | 534.0 | 1,080 | 5,435 | 5,271 |
|  | (12,334 - 13,202) | (506.5 - 561.4) | (966.9 - 1,194) | (5,167 - 5,703) | (5,002 - 5,541) |
| Difference ^c^ | **-****9544***** | **-****430.6***** | **-****3325***** | **-3491***** | **-2819***** |
|  | **(****-10112 to -8975)** | **(-****467.9 to -393.3)** | **(-****3581 to -3070)** | **(-3812 to -3169)** | **(-3192 to -2447)** |
| Observations | 128,528 | 128,528 | 128,528 | 128,528 | 128,528 |
| Non-Hispanic White |  |  |  |  |  |
| Multi-pill therapy | 22,554 | 935.0 | 4,103 | 8,661 | 9,022 |
|  | (22,143 - 22,964) | (905.6 - 964.4) | (3,827 - 4,379) | (8,441 - 8,881) | (8,719 - 9,325) |
| Combination-pill therapy | 13,865 | 504.5 | 1,232 | 5,426 | 6,270 |
|  | (13,089 - 14,641) | (463.3 - 545.6) | (1,039 - 1,425) | (4,965 - 5,887) | (5,766 - 6,773) |
| Difference | **-8688***** | **-430.5***** | **-2872***** | **-3235***** | **-2752***** |
|  | **(-****9590 to -7787)** | **(-480.7 to -380.3)** | **(-3190 to -2553)** | **(-3750 to -2719)** | **(-3351 to -2153)** |
| Observations | 60,139 | 60,139 | 60,139 | 60,139 | 60,139 |
| Non-Hispanic Black |  |  |  |  |  |
| Multi-pill therapy | 24,594 | 1,145 | 4,855 | 10,616 | 7,959 |
|  | (23,939 - 25,248) | (1,106 - 1,185) | (4,488 - 5,221) | (10,290 - 10,943) | (7,507 - 8,411) |
| Combination-pill therapy | 12,861 | 638.7 | 928.2 | 6,208 | 4,624 |
|  | (12,181 - 13,540) | (587.5 - 689.8) | (777.8 - 1,079) | (5,748 - 6,668) | (4,240 - 5,007) |
| Difference | **-****11733***** | **-506.7***** | **-3926***** | **-4408***** | **-3335***** |
|  | **(-****12667 to -10798)** | **(-572.2 to -441.3)** | **(-4313 to -3529)** | **(-4976 to -3841)** | **(-3892 to -2778)** |
| Observations | 39,326 | 39,326 | 39,326 | 39,326 | 39,326 |
| Hispanic |  |  |  |  |  |
| Multi-pill therapy | 11,679 | 297.5 | 2,378 | 4,076 | 4,831 |
|  | (11,114 - 12,244) | (266.8 - 328.2) | (2,063 - 2,693) | (3,790 - 4,362) | (4,444 - 5,219) |
| Combination-pill therapy | 6,630 | 159.6 | 523.4 | 2,433 | 3,181 |
|  | (5,703 - 7,558) | (127.2 - 192.0) | (286.9 - 759.9) | (2,052 - 2,814) | (2,456 - 3,905) |
| Difference | **-****5048***** | **-137.9***** | **-1855***** | **-1643***** | **-1650***** |
|  | **(-****6139 to -3958)** | **(-182.0 to -93.8)** | **(-2253 to -1456)** | **(-2117 to -1169)** | **(-2476 to -825.7)** |
| Observations | 8,690 | 8,690 | 8,690 | 8,690 | 8,690 |

^a^ A generalized linear model with a gamma distribution and log link was used for all cost outcomes. Average marginal effects, along with 95% confidence intervals, were reported. All models were adjusted for 12 Charlson comorbidities, risk factors (alcohol use, tobacco use, obesity, and lipid disorders), gender, age groups (aged 18-34, aged 35-44, aged 45-54, and aged 55-64), race/ethnicity, and fixed effects for the year and month of the index date.

^b^ FDC comprises individuals who exclusively used FDC during the one-year follow-up periods from the antihypertensive index date. Multi-pill therapy comprises those who did not use FDC during the one-year follow-up periods.

^c^ The reported differences reflect the predicted outcomes, specifically the average marginal effects, for individuals receiving combination-pill therapy compared with those receiving multi-pill therapy.

*** *P*<0.001, ** *P*<0.01, * *P*<0.05

**Supplemental Table 5. Sensitivity Analysis: Summary Statistics**

|  | All | Combination-pill therapy | Multi-pill therapy | P-values |
| --- | --- | --- | --- | --- |
|  | N=378,398  (100%) | N=82,126  (22%) | N=296,272  (78%) |  |
| Age, mean (SD) | 49.3 (10.1) | 49.1 (9.6) | 49.4 (10.2) | <0.001 |
| Age groups, n (%) |  |  |  |  |
| 18-34 | 35,383 (9.35%) | 6,839 (8.33%) | 28,544 (9.63%) | <0.001 |
| 35-44 | 79,225 (20.94%) | 19,109 (23.27%) | 60,116 (20.29%) | <0.001 |
| 45-54 | 120,352 (31.81%) | 27,094 (32.99%) | 93,258 (31.48%) | <0.001 |
| 55-64 | 143,438 (37.91%) | 29,084 (35.41%) | 114,354 (38.60%) | <0.001 |
| Female, n (%) | 214,147 (56.59%) | 51,173 (62.31%) | 162,974 (55.01%) | <0.001 |
| Race categories, n (%) |  |  |  |  |
| Non-Hispanic White | 198,297 (52.40%) | 38,518 (46.90%) | 159,779 (53.93%) | <0.001 |
| Non-Hispanic Black | 113,359 (29.96%) | 29,949 (36.47%) | 83,410 (28.15%) | <0.001 |
| Hispanic | 15,324 (4.05%) | 2,878 (3.50%) | 12,446 (4.20%) | <0.001 |
| Other race groups | 51,418 (13.59%) | 10,781 (13.13%) | 40,637 (13.72%) | <0.001 |
| Risk factors, n (%) |  |  |  |  |
| Alcohol use | 23,616 (6.24%) | 3,145 (3.83%) | 20,471 (6.91%) | <0.001 |
| Tobacco use | 95,337 (25.19%) | 17,001 (20.70%) | 78,336 (26.44%) | <0.001 |
| Obesity | 93,608 (24.74%) | 20,540 (25.01%) | 73,068 (24.66%) | 0.041 |
| Lipid disorders | 119,540 (31.59%) | 23,224 (28.28%) | 96,316 (32.51%) | <0.001 |
| Comorbidities, n (%) |  |  |  |  |
| Dementia | 1,659 (0.44%) | 178 (0.22%) | 1,481 (0.50%) | <0.001 |
| Chronic pulmonary disease | 81,708 (21.59%) | 14,416 (17.55%) | 67,292 (22.71%) | <0.001 |
| Rheumatic disease | 7,836 (2.07%) | 1,458 (1.78%) | 6,378 (2.15%) | <0.001 |
| Peptic ulcer disease | 2,578 (0.68%) | 322 (0.39%) | 2,256 (0.76%) | <0.001 |
| Mild liver disease | 21,747 (5.75%) | 2,815 (3.43%) | 18,932 (6.39%) | <0.001 |
| Diabetes without chronic complication | 117,737 (31.11%) | 21,427 (26.09%) | 96,310 (32.51%) | <0.001 |
| Diabetes with chronic complication | 46,081 (12.18%) | 6,685 (8.14%) | 39,396 (13.30%) | <0.001 |
| Hemiplegia or paraplegia | 5,206 (1.38%) | 617 (0.75%) | 4,589 (1.55%) | <0.001 |
| Any malignancy | 11,512 (3.04%) | 2,035 (2.48%) | 9,477 (3.20%) | <0.001 |
| Moderate or severe liver disease | 3,202 (0.85%) | 157 (0.19%) | 3,045 (1.03%) | <0.001 |
| Metastatic solid tumor | 1,914 (0.51%) | 279 (0.34%) | 1,635 (0.55%) | <0.001 |
| AIDS/HIV | 3,588 (0.95%) | 676 (0.82%) | 2,912 (0.98%) | <0.001 |

Abbreviations:

**Supplemental Table 6. Sensitivity analysis: The Association of Ever Using Combination-Pill Therapy with Medication Adherence and Medication Possession Ratios (per individual)^a^**

|  | All | NH White | NH Black | Hispanic |
| --- | --- | --- | --- | --- |
| Medication Adherence to Antihypertensives | | | | |
| Multi-pill therapy ^b^ | 0.403 | 0.453 | 0.324 | 0.390 |
|  | (0.401 - 0.405) | (0.450 - 0.455) | (0.321 - 0.328) | (0.381 - 0.399) |
| Ever using combination-pill therapy ^b^ | 0.451 | 0.510 | 0.359 | 0.426 |
|  | (0.448 - 0.454) | (0.505 - 0.515) | (0.353 - 0.364) | (0.408 - 0.444) |
| Difference ^c^ | **0.048***** | **0.057***** | **0.034***** | **0.036***** |
|  | **(0.044, 0.051)** | **(0.052,0.063)** | **(0.028, 0.04)** | **(0.016, 0.056)** |
| Observations | 378,398 | 198,297 | 113,359 | 15,324 |
|  |  |  |  |  |
| Medication Possession Ratios, % | | | | |
| Multi-pill therapy | 66.53 | 70.03 | 60.81 | 66.74 |
|  | (66.41 - 66.64) | (69.87 - 70.18) | (60.60 - 61.02) | (66.18 - 67.31) |
| Ever using combination-pill therapy | 70.19 | 74.36 | 63.36 | 70.39 |
|  | (69.98 - 70.40) | (74.06 - 74.67) | (63.01 - 63.70) | (69.30 - 71.48) |
| Difference | **3.66***** | **4.33***** | **2.55***** | **3.65***** |
|  | **(3.42,3.90)** | **(3.99,4.68)** | **(2.14,2.95)** | **(2.42,4.87)** |
| Observations | 378,398 | 198,297 | 113,359 | 15,324 |

^a^ Logistic regression was employed to analyze medication adherence, while linear regression was used for medication possession ratios. Average marginal effects, along with 95% confidence intervals, were reported. All models were adjusted for 12 Charlson comorbidities, risk factors (alcohol use, tobacco use, obesity, and lipid disorders), gender, age groups (aged 18-34 [reference], aged 35-44, aged 45-54, and aged 55-64), and fixed effects for the year and month of the index date.

^b^ Ever using combination-pill therapy comprise individuals who used any FDC during the one-year follow-up periods from the antihypertensive index date. Multi-pill therapy comprise those who did not use FDC during the one-year follow-up periods.

^c^ The reported differences reflect the predicted outcomes, specifically the average marginal effects, for individuals receiving combination-pill therapy compared with those receiving multi-pill therapy.

*** *P*<0.001, ** *P*<0.01, * *P*<0.05

**Supplemental Table 7. Sensitivity analysis: The association of ever using combination-pill therapy with health care utilization (per 1,000 individuals)^a^**

|  | Num. ED visits | Num. Inpatient Admissions | Avg. LOS | Total LOS |
| --- | --- | --- | --- | --- |
| All |  |  |  |  |
| Multi-pill therapy ^b^ | 1,552 | 366.9 | 1,035 | 2,248 |
|  | (1,539 - 1,565) | (361.1 - 372.7) | (1,015 - 1,056) | (2,187 - 2,308) |
| Ever using combination-pill therapy ^b^ | 1,308 | 213.7 | 602.2 | 1,109 |
|  | (1,290 - 1,326) | (208.6 - 218.7) | (583.8 - 620.6) | (1,067 - 1,150) |
| Difference ^c^ | **-243.8***** | **-153.2***** | **-433.0***** | **-1139***** |
|  | **(-264.6 to -223.0)** | **(-160.7 to -145.7)** | **(-461.1 to -404.9)** | **(-1209 to -1068)** |
| Observations | 378,398 | 378,398 | 378,398 | 378,398 |
| Non-Hispanic White |  |  |  |  |
| Multi-pill therapy | 1,462 | 356.6 | 997.7 | 2,126 |
|  | (1,446 - 1,478) | (349.7 - 363.6) | (972.8 - 1,023) | (2,057 - 2,196) |
| Ever using combination-pill therapy | 1,213 | 220.1 | 611.1 | 1,128 |
|  | (1,189 - 1,237) | (212.6 - 227.6) | (584.3 - 637.9) | (1,065 - 1,190) |
| Difference | **-248.6***** | **-136.5***** | **-386.6***** | **-998.7***** |
|  | **(-276.5 to -220.7)** | **(-146.5 to -126.6)** | **(-423.3 to -349.9)** | **(-1090 to -907.3)** |
| Observations | 198,297 | 198,297 | 198,297 | 198,297 |
| Non-Hispanic Black |  |  |  |  |
| Multi-pill therapy | 1,743 | 401.5 | 1,159 | 2,605 |
|  | (1,719 - 1,766) | (391.6 - 411.5) | (1,119 - 1,198) | (2,501 - 2,709) |
| Ever using combination-pill therapy | 1,487 | 208.3 | 606.2 | 1,121 |
|  | (1,456 - 1,519) | (199.5 - 217.0) | (573.4 - 639.0) | (1,050 - 1,192) |
| Difference | **-255.0***** | **-193.3***** | **-552.5***** | **-1484***** |
|  | **(-293.2 to -216.9)** | **(-206.4 to -180.1)** | **(-604.2 to -500.8)** | **(-1607 to -1361)** |
| Observations | 113,359 | 113,359 | 113,359 | 113,359 |
| Hispanic |  |  |  |  |
| Multi-pill therapy | 1,300 | 225.0 | 695.6 | 1,291 |
|  | (1,253 - 1,347) | (210.8 - 239.2) | (624.5 - 766.7) | (1,148 - 1,434) |
| Ever using combination-pill therapy | 1,119 | 159.9 | 464.3 | 822.2 |
|  | (1,045 - 1,193) | (137.3 - 182.6) | (383.2 - 545.4) | (651.5 - 992.9) |
| Difference | **-181.5***** | **-65.05***** | **-231.2***** | **-468.8***** |
|  | **(-269.1 to -93.91)** | **(-91.78 to -38.32)** | **(-339.4 to -123.1)** | **(-691.0 to -246.6)** |
| Observations | 15,324 | 15,324 | 15,324 | 15,324 |

^a^ A negative binomial model was used for all count variables. Average marginal effects, along with 95% confidence intervals, were reported. All models were adjusted for 12 Charlson comorbidities, risk factors (alcohol use, tobacco use, obesity, and lipid disorders), gender, age groups (aged 18-34 [reference], aged 35-44, aged 45-54, and aged 55-64), and fixed effects for the year and month of the index date.

^b^ Ever using combination-pill therapy comprise individuals who used any FDC during the one-year follow-up periods from the antihypertensive index date. Multi-pill therapy comprise those who did not use FDC during the one-year follow-up periods.

^c^ The reported differences reflect the predicted outcomes, specifically the average marginal effects, for individuals receiving combination-pill therapy compared with those receiving multi-pill therapy.

*** *P*<0.001, ** *P*<0.01, * *P*<0.05

**Supplemental Table 8. The association of ever using combination-pill therapy with hypertension- and CVD-related health care utilization (per 1,000 individuals)^a^**

|  | Num. HTN-related^c^ ED visits | Num. HTN-related Inpatient Admissions | Num. CVD-related^d^ ED visits | Num. CVD-related Inpatient Admissions |
| --- | --- | --- | --- | --- |
| All |  |  |  |  |
| Multi-pill therapy ^b^ | 761.2 | 277.2 | 843.1 | 319.0 |
|  | (753.4 - 768.9) | (272.6 - 281.7) | (834.8 - 851.4) | (313.8 - 324.2) |
| Combination-pill therapy ^b^ | 702.0 | 174.3 | 744.6 | 189.7 |
|  | (690.5 - 713.4) | (170.0 - 178.7) | (732.6 - 756.5) | (185.0 - 194.3) |
| Differences ^c^ | **-59.21***** | **-102.8***** | **-98.51***** | **-129.3***** |
|  | **(-72.40 to -46.02)** | **(-109.0 to -96.71)** | **(-112.3 to -84.66)** | **(-126.2 to -122.5)** |
| Observations | 378,398 | 378,398 | 378,398 | 378,398 |
| Non-Hispanic White |  |  |  |  |
| Multi-pill therapy | 638.9 | 249.0 | 723.6 | 296.9 |
|  | (630.1 - 647.6) | (243.9 - 254.1) | (714.0 - 733.2) | (290.9 - 302.8) |
| Combination-pill therapy | 599.3 | 171.9 | 643.9 | 189.7 |
|  | (584.5 - 614.1) | (165.7 - 178.1) | (628.3 - 659.4) | (183.0 - 196.5) |
| Differences | **-39.55***** | **-77.08***** | **-79.75***** | **-107.1***** |
|  | **(-56.43 to -22.67)** | **(-84.98 to -69.19)** | **(-97.62 to -61.88)** | **(-116.0 to -98.25)** |
| Observations | 198,297 | 198,297 | 198,297 | 198,297 |
| Non-Hispanic Black |  |  |  |  |
| Multi-pill therapy | 963.4 | 329.9 | 1,046 | 366.2 |
|  | (948.4 - 978.4) | (321.5 - 338.3) | (1,030 - 1,062) | (357.0 - 375.5) |
| Combination-pill therapy | 870.1 | 177.7 | 912.2 | 190.4 |
|  | (849.1 - 891.1) | (170.0 - 185.4) | (890.5 - 933.8) | (182.2 - 198.6) |
| Differences | **-93.27***** | **-152.1***** | **-134.3***** | **-175.8***** |
|  | **(-118.5 to -68.01)** | **(-163.5 to -140.8)** | **(-160.5 to -108.0)** | **(-188.1 to -163.5)** |
| Observations | 113,359 | 113,359 | 113,359 | 113,359 |
| Hispanic |  |  |  |  |
| Multi-pill therapy | 552.4 | 166.2 | 612.7 | 192.5 |
|  | (527.6 - 577.2) | (154.9 - 177.4) | (586.4 - 639.0) | (179.8 - 205.1) |
| Combination-pill therapy | 547.7 | 139.9 | 580.4 | 146.8 |
|  | (497.4 - 598.1) | (118.6 - 161.1) | (528.3 - 632.4) | (124.8 - 168.8) |
| Differences | **-4.64** | **-26.32*** | **-32.32** | **-45.64***** |
|  | **(-60.53 to 51.24)** | **(-50.4 to -2.24)** | **(-90.43 to 25.79)** | **(-71.03 to -20.25)** |
| Observations | 15,324 | 15,324 | 15,324 | 15,324 |

^a^ A negative binomial model was used for all count variables. Average marginal effects, along with 95% confidence intervals, were reported. All models were adjusted for 12 Charlson comorbidities, risk factors (alcohol use, tobacco use, obesity, and lipid disorders), gender, age groups (aged 18-34 [reference], aged 35-44, aged 45-54, and aged 55-64), and fixed effects for the year and month of the index date.

^b^ Ever using combination-pill therapy comprise individuals who used any FDC during the one-year follow-up periods from the antihypertensive index date. Multi-pill therapy comprise those who did not use FDC during the one-year follow-up periods.

^c^ The reported differences reflect the predicted outcomes, specifically the average marginal effects, for individuals receiving combination-pill therapy compared with those receiving multi-pill therapy.

^d^ Hypertension (HTN)-related emergency department (ED) visits and inpatient admissions are defined as those containing an HTN diagnosis (ICD-10-CM codes I10–I15). Cardiovascular disease (CVD)-related ED visits and inpatient admissions are defined as those containing a CVD diagnosis (ICD-10-CM codes I00–I78).

*** *P*<0.001, ** *P*<0.01, * *P*<0.05

**Supplemental Table 9. Sensitivity analysis: The association of any combination-pill therapy use with medical costs per individual^a^**

|  | Total medical costs, $ | ED costs, $ | Inpatient costs, $ | Outpatient costs, $ | Pharmacy prescription costs, $ |
| --- | --- | --- | --- | --- | --- |
| All |  |  |  |  |  |
| Multi-pill therapy^b^ | 24,860 | 1,076 | 5,175 | 9,652 | 8,711 |
|  | (24,590 - 25,131) | (1,056 - 1,096) | (5,004 - 5,346) | (9,513 - 9,790) | (8,535 - 8,887) |
| Any combination-pill therapy ^b^ | 18,440 | 829.5 | 2,734 | 7,193 | 7,262 |
|  | (18,092 - 18,787) | (804.6 - 854.3) | (2,581 - 2,888) | (7,019 - 7,367) | (7,022 - 7,502) |
| Differences ^c^ | **-6420***** | **-246.5***** | **-2440***** | **-2458***** | **-1449** |
|  | **(-6882 to -5959)** | **(-276.6 to -216.5)** | **(-2677 to -2205)** | **(-2687 to -2229)** | **(-1772 to -1126)** |
| Observations | 148,121 | 148,121 | 148,121 | 148,121 | 148,121 |
| Non-Hispanic White |  |  |  |  |  |
| Multi-pill therapy | 25,093 | 1,037 | 4,751 | 9,243 | 9,871 |
|  | (24,736 - 25,449) | (1,011 - 1,063) | (4,550 - 4,951) | (9,055 - 9,430) | (9,637 - 10,105) |
| Any combination-pill therapy | 19,481 | 769.9 | 2,893 | 7,014 | 8,485 |
|  | (18,843 - 20,120) | (730.8 - 809.1) | (2,609 - 3,177) | (6,723 - 7,305) | (7,985 - 8,986) |
| Differences | **-5611***** | **-267.0***** | **-1858***** | **-2229***** | **-1385***** |
|  | **(-6367 to -4855)** | **(-312.3 to -221.6)** | **(-2208 to -1508)** | **(-2583 to -1874)** | **(-1974 to -796.5)** |
| Observations | 67,484 | 67,484 | 67,484 | 67,484 | 67,484 |
| Non-Hispanic Black |  |  |  |  |  |
| Multi-pill therapy | 27,123 | 1,243 | 5,687 | 11,497 | 8,332 |
|  | (26,602 - 27,644) | (1,209 - 1,277) | (5,387 - 5,987) | (11,220 - 11,773) | (8,022 - 8,642) |
| Any combination-pill therapy | 18,877 | 945.6 | 2,531 | 8,256 | 6,598 |
|  | (18,352 - 19,402) | (906.7 - 984.4) | (2,308 - 2,754) | (7,959 - 8,554) | (6,293 - 6,903) |
| Differences | **-8246***** | **-297.5***** | **-3156***** | **-3240***** | **-1734***** |
|  | **(-8999 to -7493)** | **(-347.9 to -247.0)** | **(-3532 to -2781)** | **(-3650 to -2829)** | **(-2177 to -1291)** |
| Observations | 47,666 | 47,666 | 47,666 | 47,666 | 47,666 |
| Hispanic |  |  |  |  |  |
| Multi-pill therapy | 12,758 | 313.6 | 2,649 | 4,295 | 5,235 |
|  | (12,223 - 13,293) | (284.9 - 342.3) | (2,350 - 2,948) | (4,040 - 4,550) | (4,887 - 5,583) |
| Any combination-pill therapy | 9,689 | 266.3 | 1,447 | 3,053 | 4,607 |
|  | (8,909 - 10,470) | (229.7 - 303.0) | (1,134 - 1,761) | (2,764 - 3,343) | (3,961 - 5,252) |
| Differences | **-3068***** | **-47.27*** | **-1202***** | **-1241***** | **-628.5** |
|  | **(-4017 to -2120)** | **(-93.7 to -0.83)** | **(-1634 to -769.4)** | **(-1627 to -855.8)** | **(-1368 to 110.5)** |
| Observations | 9,580 | 9,580 | 9,580 | 9,580 | 9,580 |

^a^ A generalized linear model with a gamma distribution and log link was used for all cost outcomes. Average marginal effects, along with 95% confidence intervals, were reported. All models were adjusted for 12 Charlson comorbidities, risk factors (alcohol use, tobacco use, obesity, and lipid disorders), gender, age groups (aged 18-34 [reference], aged 35-44, aged 45-54, and aged 55-64), and fixed effects for the year and month of the index date.

^b^ Ever using combination-pill therapy comprise individuals who used any FDC during the one-year follow-up periods from the antihypertensive index date. Multi-pill therapy comprise those who did not use FDC during the one-year follow-up periods.

^c^ The reported differences reflect the predicted outcomes, specifically the average marginal effects, for individuals receiving combination-pill therapy compared with those receiving multi-pill therapy.

*** *P*<0.001, ** *P*<0.01, * *P*<0.05

**Supplemental Table 10. The Association of Combination-Pill Therapy Use with Medication Adherence and Medication Possession Ratios (per individual)^a^, Unweighted**

|  | All | NH White | NH Black | Hispanic |
| --- | --- | --- | --- | --- |
| Medication Adherence (MPR ≥ 80%) to Antihypertensives | | | | |
| Multi-pill therapy ^b^ | 0.411 | 0.452 | 0.331 | 0.389 |
|  | (0.409 - 0.413) | (0.450 - 0.454) | (0.327 - 0.334) | (0.381 - 0.398) |
| Combination-pill therapy only ^b^ | 0.495 | 0.549 | 0.390 | 0.448 |
|  | (0.490 - 0.501) | (0.542 - 0.557) | (0.381 - 0.400) | (0.422 - 0.475) |
| Difference^c^ | **0.085***** | **0.097***** | **0.060***** | **0.059***** |
|  | **(0.079 - 0.091)** | **(0.089 - 0.106)** | **(0.050 - 0.070)** | **(0.031 - 0.087)** |
| Observations | 325,600 | 174,306 | 93,122 | 13,736 |
|  |  |  |  |  |
| Medication Possession Ratios | | | | |
| Multi-pill therapy | 67.24 | 70.13 | 61.43 | 66.86 |
|  | (67.13 – 67.35) | (69.98 – 70.28) | (61.22 – 61.64) | (66.32 – 67.40) |
| Combination-pill therapy only | 70.34 | 74.09 | 62.31 | 70.58 |
|  | (69.98 – 70.69) | (73.59 – 74.59) | (61.69 – 62.92) | (68.91 – 72.25) |
| Difference | **3.10***** | **3.96***** | **0.87**** | **3.72***** |
|  | **(2.73 - 3.47)** | **(3.44 - 4.48)** | **(0.23 – 1.52)** | **(1.96 - 5.47)** |
| Observations | 325,600 | 174,306 | 93,122 | 13,736 |

^a^ Logistic regression was employed to analyze medication adherence, while linear regression was used for medication possession ratios. Average marginal effects, along with 95% confidence intervals, were reported. All models were adjusted for 12 Charlson comorbidities, risk factors (alcohol use, tobacco use, obesity, and lipid disorders), sex, age groups (aged 18-34, aged 35-44, aged 45-54, and aged 55-64), race/ethnicity, and fixed effects for the year and month of the index date.

^b^ Those who used combination-pill therapy comprise individuals who exclusively used FDC-antihypertensives during the one-year follow-up periods from the antihypertensive index date. Those who used multi-pill therapy comprise individuals who did not use FDC-antihypertensives during the one-year follow-up periods.

^c^ The reported differences reflect the predicted outcomes, specifically the average marginal effects, for individuals receiving combination-pill therapy compared with those receiving multi-pill therapy.

*** *P*<0.001, ** *P*<0.01, * *P*<0.05

**Supplemental Table 11. The Association of Combination-Pill Therapy Use with Hypertension- and Cardiovascular Disease- Related Health Care Utilization (per 1,000 individuals)^a^, Unweighted**

|  | Num. HTN-related ^c^ ED visits | Num. HTN-related Inpatient Admissions | Num. CVD-related^d^ ED visits | Num. CVD-related Inpatient Admissions |
| --- | --- | --- | --- | --- |
| All |  |  |  |  |
| Multi-pill therapy ^b^ | 798.5 | 328.4 | 894.9 | 382.8 |
|  | (783.1 – 813.9) | (316.2 – 340.7) | (878.3 – 911.4) | (368.7 – 396.9) |
| Combination-pill therapy only ^b^ | 552.1 | 110.5 | 585.2 | 119.9 |
|  | (521.9 – 582.4) | (100.7 – 120.4) | (554.6 – 615.8) | (109.6 – 130.1) |
| Difference^c^ | **-246.4***** | **-217.9***** | **-309.7***** | **-262.9***** |
|  | **(-278.7 – -214.1)** | **(-231.0 – -204.8)** | **(-342.5 – -276.8)** | **(-277.4 – -248.5)** |
| Observations | 325,600 | 325,600 | 325,600 | 325,600 |
| Non-Hispanic White |  |  |  |  |
| Multi-pill therapy | 686.0 | 297.8 | 785.5 | 359.0 |
|  | (669.1 – 702.9) | (284.6 – 311.0) | (767.0 – 803.9) | (343.5 – 374.6) |
| Combination-pill therapy only | 492.4 | 108.9 | 525.2 | 118.7 |
|  | (454.9 – 529.9) | (95.91 – 122.0) | (487.1 – 563.3) | (105.1 – 132.3) |
| Difference | **-193.6***** | **-188.9***** | **-260.2***** | **-240.3***** |
|  | **(-233.5 – -153.7)** | **(-205.4 – -172.3)** | **(-301.2 – -219.3)** | **(-258.6 – -222.0)** |
| Observations | 174,306 | 174,306 | 174,306 | 174,306 |
| Non-Hispanic Black |  |  |  |  |
| Multi-pill therapy | 1,035 | 406.0 | 1,134 | 453.3 |
|  | (1,002 – 1,069) | (383.7 – 428.3) | (1,099 – 1,169) | (429.2 – 477.4) |
| Combination-pill therapy only | 710.7 | 116.6 | 749.7 | 126.4 |
|  | (645.1 – 776.4) | (99.83 – 133.4) | (683.7 – 815.6) | (109.0 – 143.8) |
| Difference | **-324.5***** | **-289.4***** | **-384.6***** | **-326.9***** |
|  | **(-397.1 – -252.0)** | **(-315.4 – -263.3)** | **(-457.9 – -311.3)** | **(-354.5 – -299.4)** |
| Observations | 93,122 | 93,122 | 93,122 | 93,122 |
| Hispanic |  |  |  |  |
| Multi-pill therapy | 595.1 | 202.4 | 664.9 | 237.6 |
|  | (547.0 – 643.2) | (177.3 – 227.6) | (613.8 – 716.0) | (209.4 – 265.9) |
| Combination-pill therapy only | 456.9 | 76.58 | 477.6 | 79.58 |
|  | (342.6 – 571.1) | (47.38 – 105.8) | (364.0 – 591.2) | (50.64 – 108.5) |
| Difference | **-138.3*** | **-125.9***** | **-187.3**** | **-158.1***** |
|  | **(-262.0 – -14.47)** | **(-164.1 – -87.63)** | **(-311.7 – -62.94)** | **(-198.1 – -118.0)** |
| Observations | 13,736 | 13,736 | 13,736 | 13,736 |

^a^ A negative binomial model was used for all count variables. Average marginal effects, along with 95% confidence intervals, were reported. All models were adjusted for 12 Charlson comorbidities, risk factors (alcohol use, tobacco use, obesity, and lipid disorders), sex, age groups (aged 18-34, aged 35-44, aged 45-54, and aged 55-64), race/ethnicity and fixed effects for the year and month of the index date.

^b^ Those who used combination-pill therapy comprise individuals who exclusively used FDC-antihypertensive during the one-year follow-up periods from the antihypertensive index date. Those who used multi-pill therapy comprise those who did not use FDC-antihypertensive during the one-year follow-up periods.

^c^ The reported differences reflect the predicted outcomes, specifically the average marginal effects, for individuals receiving combination-pill therapy compared with those receiving multi-pill therapy.

^d^ Hypertension (HTN)-related emergency department (ED) visits and inpatient admissions are defined as those containing an HTN diagnosis (ICD-10-CM codes I10–I15). Cardiovascular disease (CVD)-related ED visits and inpatient admissions are defined as those containing a CVD diagnosis (ICD-10-CM codes I00–I78).

*** *P*<0.001, ** *P*<0.01, * *P*<0.05

**Supplemental Table 12. The Association of Combination-Pill Therapy Use with Medical Costs Associated with Hypertension and Cardiovascular Disease (CVD) (per individual)^a^, Unweighted**

|  | HTN-related total medical costs, $ | HTN-related ED costs, $ | HTN-related inpatient costs, $ | CVD-related total medical costs, $ | CVD-related ED costs, $ | CVD-related inpatient costs, $ |
| --- | --- | --- | --- | --- | --- | --- |
| All |  |  |  |  |  |  |
| Multi-pill therapy ^b^ | 6,539 | 543 | 4,250 | 8,008 | 610 | 5,133 |
|  | (6,216 – 6,503) | (527 - 560) | (4,088 – 4,413) | (7,834 – 8,182) | (592 - 627) | (4,940 – 5,327) |
| Combination-pill therapy only ^b^ | 2,654 | 293 | 1,178 | 2,990 | 306 | 1,317 |
|  | (2,487 – 2,821) | (268 - 318) | (1,059 – 1,298) | (2,812 – 3,168) | (281 - 331) | (1,190 – 5,327) |
| Difference ^c^ | **-3,705***** | **-250***** | **-3,072***** | **-5,018***** | **-304***** | **-3,816***** |
|  | **(-3,902 – -3,508)** | **(-278 – -223)** | **(-3,246 – -2,897)** | **(-5,238 – -4,798)** | **(-332 – -276)** | **(-4,015 – -3,617)** |
| Observations | 128,528 | 128,528 | 128,528 | 128,528 | 128,528 | 128,528 |
| Non-Hispanic White |  |  |  |  |  |  |
| Multi-pill therapy | 5,262 | 446 | 3,650 | 6,866 | 520 | 4,629 |
|  | (5,110 – 5,414) | (428 - 463) | (3,475 – 3,825) | (6,677 – 7,055) | (501 - 540) | (4,412 – 4,846) |
| Combination-pill therapy only | 2,432 | 261 | 1,268 | 2,734 | 275 | 1,433 |
|  | (2,205 – 2,659) | (228 - 294) | (1,084 - 1,451) | (2,493 – 2,975) | (242 - 308) | (1,235 – 1,632) |
| Difference | **-2,830***** | **-184***** | **-2,383***** | **-4,132***** | **-245***** | **-3,196***** |
|  | **(-3,089 – -2,571)** | **(-220 – -149)** | **(-2,616 – -2,150)** | **(-4,419 – -3,844)** | **(-282 – -209)** | **(-3,463 – -2,928)** |
| Observations | 60,139 | 60,139 | 60,139 | 60,139 | 60,139 | 60,139 |
| Non-Hispanic Black |  |  |  |  |  |  |
| Multi-pill therapy | 8,516 | 739 | 5,202 | 10,362 | 809 | 6,001 |
|  | (8,219 – 8,812) | (704 - 774) | (4,911 – 5,493) | (10,016 – 10,707) | (773 - 845) | (5,674 – 6,327) |
| Combination-pill therapy only | 3,160 | 387 | 1,014 | 3,551 | 405 | 1,141 |
|  | (2,856 – 3,465) | (337 - 438) | (862 – 1,166) | (3,228 – 3,874) | (355 - 455) | (978 – 1,305) |
| Difference | **-5,355***** | **-352***** | **-4,188***** | **-6,811***** | **-405***** | **-4,860***** |
|  | **(-5,761 – -4,950)** | **(-411 – -292)** | **(-4,499 – -3,878)** | **(-7,259 – -6,363)** | **(-464 – -345)** | **(-5,204 – -4,515)** |
| Observations | 39,326 | 39,326 | 39,326 | 39,326 | 39,326 | 39,326 |
| Hispanic |  |  |  |  |  |  |
| Multi-pill therapy | 2,603 | 107 | 2,148 | 3,343 | 120 | 2,685 |
|  | (2,425 – 2,780) | (97 - 117) | (1,921 – 2,376) | (3,128 – 3,558) | (109 - 130) | (2,413 – 2,957) |
| Combination-pill therapy only | 1,000 | 60 | 603 | 1,086 | 64 | 638 |
|  | (790 – 1,209) | (43 - 77) | (408 - 797) | (872 – 1,301) | (46 - 81) | (442 - 834) |
| Difference | **-1,603***** | **-47***** | **-1,546***** | **-2,256***** | **-56***** | **-2,047***** |
|  | **(-1,876 – -1,330)** | **(-67 – -27)** | **(-1,842 – -1,249)** | **(-2,558 – -1,955)** | **(-76 – -36)** | **(-2,379 – -1,715)** |
| Observations | 8,690 | 8,690 | 8,690 | 8,690 | 8,690 | 8,690 |

^a^ Hypertension-related and CVD-related healthcare utilization and total medical costs were defined based on the presence of respective diagnosis codes (hypertension: ICD-10-CM codes I10-I15; CVD: ICD-10-CM codes I00-I78). A generalized linear model with a gamma distribution and log link was used for all cost outcomes. Average marginal effects, along with 95% confidence intervals, were reported. All models were adjusted for 12 Charlson comorbidities, risk factors (alcohol use, tobacco use, obesity, and lipid disorders), sex, age groups (aged 18-34, aged 35-44, aged 45-54, and aged 55-64), race/ethnicity, and fixed effects for the year and month of the index date.

^b^ Those who used combination-pill therapy comprise individuals who exclusively used FDC-antihypertensives during the one-year follow-up periods from the antihypertensive index date. Those who used multi-pill therapy comprise those who did not use FDC-antihypertensive during the one-year follow-up periods.

^c^ The reported differences reflect the predicted outcomes, specifically the average marginal effects, for individuals receiving combination-pill therapy compared with those receiving multi-pill therapy.

*** *P*<0.001, ** *P*<0.01, * *P*<0.05
